# Filling the Knowledge Gap: Measuring HIV Prevalence and Risk Factors among Men Who Have Sex with Men and Female Sex Workers in Tripoli, Libya

**DOI:** 10.1371/journal.pone.0066701

**Published:** 2013-06-19

**Authors:** Joseph J. Valadez, Sima Berendes, Caroline Jeffery, Joanna Thomson, Hussain Ben Othman, Leon Danon, Abdullah A. Turki, Rabea Saffialden, Lusine Mirzoyan

**Affiliations:** 1 Liverpool School of Tropical Medicine, Pembroke Place, Liverpool, United Kingdom; 2 Mathematics Institute, University of Warwick, Coventry, United Kingdom; 3 National Centre for Diseases Control, National AIDS Programme, Tripoli, Libya; National Institute of Allergy and Infectious Diseases, United States of America

## Abstract

**Background:**

Publications on Libya’s HIV epidemic mostly examined the victims of the tragic nosocomial HIV outbreak in the 1990s and the related dispute about the detention of foreign medical workers. The dispute resolution in 2003 included an agreement with the European Union on humanitarian cooperation and the development of Libya’s first National HIV Strategy. As part of this we conducted Libya’s first bio-behavioural survey among men having sex with men (MSM) and female sex workers (FSW).

**Methods:**

Using respondent-driven sampling, we conducted a cross-sectional study to estimate the prevalence of HIV, hepatitis B virus (HBV), hepatitis C virus (HCV), and related risk factors among 227 MSM and 69 FSW in Tripoli (FSW recruitment ended prematurely due to the political events in 2011).

**Results:**

For MSM we estimated an HIV prevalence of 3.1%, HBV prevalence of 2.9%, and HCV prevalence of 7.3%, and for FSW an HIV prevalence of 15.7%, HBV prevalence of 0%, and HCV prevalence of 5.2%. We detected high levels of risk behaviours, poor HIV-related knowledge, high stigma and lack of prevention programmes. These results must be interpreted in the context of the political situation which prohibited reaching an ideal sample size for FSW.

**Conclusion:**

There is urgent need to implement an effective National HIV Strategy informed by the results of this research. The risk of transmission within different risk groups and to the general population may be high given the recent military events that led to increased violence, migration, and the disruption of essential HIV-related services.

## Introduction

An assessment of the HIV prevalence, related risk behaviour and knowledge among men who have sex with men (MSM) and female sex workers (FSW) in Libya is urgently needed to inform near-term policy making while the window of opportunity to act is still open [1], [2]. History has shown that timely collection of necessary information for the design of effective HIV prevention strategies is crucial, in order to confine the epidemic at an early stage [1]. This is particularly important in Libya which has not had a National HIV Strategy, and may face an expansion of the HIV epidemic due to increased migration, violence, and the disruption of services and structures related to recent warfare and socio-political transition [3], [4], [5].

Male-to-male sexual contact and transactional heterosexual intercourse have been main modes of HIV transmission in the early stages of the HIV epidemic in many countries [6], [7]. Recently, new emergence and resurgence of HIV infection among MSM were observed in many parts of the world [7], [8], [9]. Similarly, FSW continue to be disproportionally affected by HIV globally in almost all settings [6]. Male to male sexual intercourse, which exists in diverse cultures and throughout history, is highly stigmatized in many parts of the world, and often criminalized, especially in low and middle income countries [7], [9]. Likewise, commercial sex is illegal in most nations and severely punished in many [10]. In Libya, both MSM and sex workers have been highly stigmatized, facing corporal punishment and sentences of up to several years in prison if arrested and sentenced [11]. This behaviour creates incentives for these groups to remain hidden, resulting in an apparent lack of knowledge on HIV prevention and frequent inability to access HIV related services and tools. It might also be responsible for the striking lack of HIV-related data among these populations [2], [6], [9], [10].

Libya’s HIV epidemic has been classified as possibly concentrated among people who inject drugs (PWID) [12], which we recently confirmed [13]. (A concentrated epidemic is defined as HIV prevalence below 1% in the general population, as measured in pregnant women in urban areas, and consistently above 5% in at least one subpopulation, such as PWID [14]). However, no data exist on the HIV prevalence among MSM and FSW [12]. Sound evidence on the prevalence of Hepatitis B virus (HBV) and Hepatitis C Virus (HCV) infection among these groups is also missing [15].

So far the few international publications that exist on the HIV epidemic in Libya are mostly restricted to those examining the victims of the tragic outbreak of nosocomial infections in the 1990s [16]. The related detention of six foreign medical workers and their trials in Libya had led to international political tensions, which were only resolved in 2003 after the detainees were released and an agreement was made with the European Union on humanitarian cooperation [17], [18]. This included the development of Libya’s first National HIV Strategy, as part of which we conducted a Bio-Behavioral Surveillance Survey (BBSS) among most vulnerable populations (MVPs). We used the method of respondent-driven sampling (RDS), which has been successfully applied in many settings to sample hidden populations [19], [20], [21].

The study reported here provides baseline information on the level of HIV infection and related socio-demographic, behavioural and other characteristics among MSM and FSW in Tripoli to inform the design and future evaluation of Libya’s first National HIV Strategy.

## Methods

### Study Design and Participants

We conducted a cross-sectional BBSS among MSM and FSW in Tripoli, Libya. Given the hidden nature of these populations, we used the peer-driven chain-referral method of RDS to access study participants through their social networks [20], [21].

MSM were eligible to join the study if they were born male, and had anal sex with another man in the last six months. FSW were eligible if they were born female, and had earned all or part of their income through the exchange of money for vaginal or anal sex with more than one client in the last six months. In addition, participants of both groups had to be ≥15 years old, and have a valid referral coupon.

### Study Procedures

The study began on 21-Sep-2010 at a single site, with opening times differing for MSM and FSW, as they preferred not to share the same waiting room. Sampling started with initial participants, referred to as *seeds*, which were identified during an initial mapping exercise, carried out by an international consultant on social mapping to explore the situation, sensitize the populations of interest and identify their most active representatives as potential seeds. The criteria we used to select the seeds included clear understanding of the study goal and enthusiasm for it; good communication skills, a large personal network size and enjoying great respect among peers were assets. Because some of the seeds failed to successfully initiate recruitment chains due to the highly stigmatizing environment, we had to recruit a total of 14 MSM and 13 FSW seeds. The intensive outreach work continued even after the start of the survey. We made every effort to include seeds of diverse sociodemographic background and serostatus, as well as places of gathering. After undergoing the survey process themselves, the seeds initiated the recruitment chains by distributing up to three recruitment coupons to their peers and referring them to the study site. Eligible participants underwent a face-to-face interview, received pre-test counselling, provided blood for lab tests, had the opportunity to be seen by a doctor for sexually transmitted infections (STI), and were given the option to obtain HIV rapid test results and post-test counselling during the same, and HBV and HCV results during a second visit. They were then given a primary compensation payment to cover part of their time and transportation for the completed visit, as well as up to three coupons for peer recruitment. A secondary compensation was provided at a later stage for each successfully recruited peer.

MSM recruitment continued until a sample size of 227 was reached. This corresponds to the sample size required when using the formula proposed by Salganik et al 2006 [22] with the following parameter settings: a standard error of 0.03, a design effect of 2.5, which is higher and thus more conservative than the recommended value of 2.0, and an assumed HIV prevalence of 9%. We chose this last estimate based on the highest known prevalence in neighbouring countries [23]. We applied these same principles to FSW. With the same formula and highest known regional prevalence of 13% [24], we required a sample size of 314 FSW. However, FSW recruitment progressed much more slowly than for MSM, and was interrupted by the political events of February 2011 before the target sample size could be met [22].

### Measures

The interviews were guided by a structured questionnaire that included questions on socio-demographics, sexual and other risk behaviours, HIV-related knowledge, access to services for HIV prevention and care, and other HIV-related factors. Questions were based on internationally recognized and standardized indicators and instruments [25], [26] that were adapted to the local context. We also included RDS-specific questions on personal network size, and reciprocity of the relationship between recruiter and recruit [20], [21], [27], [28]. The questionnaires were translated into Libyan colloquial Arabic and French, back-translated into English for verification, and refined during pre-testing.

Venous blood samples taken by qualified nurses were immediately tested for HIV by laboratory technicians who applied a three serial rapid testing strategy [29] using Determine HIV-1/2 Kit (Inverness Medical Innovations) for initial testing, and subsequently Uni-Gold HIV-Kit (Trinity Biotech) and Bioline HIV-1/2-Kit (Standard Diagnostics) for confirmation of reactive results.

HBV and HCV tests were performed daily at a central reference laboratory located at the National Centre for Disease Control (NCDC) in Tripoli. After detecting HBV surface antigen (HBsAg) and HCV antibodies by automated microparticle enzyme immunoassay (MEIA, AxSym, Abbot Diagnostics), positive results were followed up with enzyme linked fluorescent assay to determine HBsAg and anti-HBV core antigen (VIDAS, Biomérieux) and with line-immunoassay to confirm anti-HCV (INNO-LIA HCV-AbIII, Innogenetics).

### Data Analysis

After double data entry using EpiInfo v.3.5.1 and data pre-processing in MS access and excel v.2012, we developed programmes in MATLAB v.2010b for statistical analysis. All seeds were excluded from the analysis. We followed current RDS methodology which assumes that RDS chains of sufficient length are Markovian, [20], [30]. We constructed the transition matrix *A* from the referral chains, where each element *a_ij_* represents the proportion of the sample with characteristic *i* that recruit individuals with characteristic *j*. Using *A* and assuming that the characteristic of the recruit is only dependent on the characteristic of the recruiter (i.e. Markovian) we were able to estimate the expected sample proportions at *equilibrium*. To confirm that the sample was not biased by the selection of seeds, which are not necessarily randomly chosen, we computed whether *equilibrium* had been reached for all relevant indicators. We define *equilibrium* to be the point at which the sample proportion for the specific indicator changes minimally with continued recruitment, and is sufficiently close to the proportion expected from a simple Markov process [20]. We distinguish *equilibrium* at 1%, where the mean absolute discrepancy between the sample proportion and the expected value from the recruitment matrix is <1%, from *equilibrium* at 2%, which results in a lower confidence. If the sample proportion is >2% different from the expected values, we conclude that the sampling process has not reached *equilibrium* for that indicator.

We used three methods to analyse the RDS chains (RDSI/DS, RDSII and RDSII/DA [20], [30]) and ensure that the representative population proportion estimates are robust. We used three methods to analyse the RDS chains (RDSI/DS, RDSII and RDSII/DA [20], [30]) to ensure that the representative population proportion estimates are robust. The first (denoted RDSI/DS), involves deriving a matrix of smoothed adjusted transition probabilities by adjusting the transition matrix *A* to account for differences in recruitment success. Using this matrix, the personal network size and the populations proportion estimates of each characteristic, *pi*, we establish a set of equations to solve for *pi* [20]. With the second method (denoted RDSII), the sample proportions of individuals in each characteristic group are weighted by the ratio between the average network sizes from the total population and from the characteristic group. This ignores the variation in recruitment success rate between individuals with different characteristics [30]. The third estimator (denoted RDSII/DA), accounts for biases that are introduced with differences in the success of recruitment of different groups by weighting the *equilibrium* proportion, rather than the sample proportion, by the same ratio of network sizes [30].

Confidence intervals were estimated using a re-sampling procedure with 10,000 reiterations [22] for all three methods. Further details on the computation of population proportion estimates are given elsewhere [13].

For the MSM data, we identified statistically significant correlates of HIV infection by calculating odds ratios using Fisher’s central exact test. The analysis was conducted in R v.2.13.1 and the packages *epitools* v.05-7 and *exact2x2* v.1.1-1.0.

### Ethics Statement

The study, including the consent procedure, was approved by the Liverpool School of Tropical Medicine Research Ethics Committee and the Libyan National Ethics Review Committee, and endorsed by the Libyan Ministry of Security, who ordered police to refrain from raids against MSM and FSW at and near the study site. Because respondents were reluctant to provide their signature for fear of being identified, we obtained oral rather than written informed consent from all study participants (having a staff member sign a certificate confirming that consent was fully informed and given freely and voluntarily). The informed consent form was administered by the screener, and the whole process was monitored by the supervisor. Informed consent forms, questionnaires and laboratory results were linked through code numbers rather than personal identifying information to ensure complete anonymity. The ethics committees also waived the need for informed consent from the next of kin, caretakers, or guardians on behalf of respondents aged 15–17, because the right for confidentiality in such a sensitive survey prevailed over the need for consent from their adult family members. This was the principle proffered by the Libyan NCDC.

Study participants who tested positive for HIV, HBV or HCV were referred to appropriate public health facilities for free clinical evaluation and treatment if needed.

## Results

We recruited a total of 227 eligible MSM through up to 15 recruitment waves and 69 FSW through 10 waves. For the MSM data, *equilibrium* was reached for all but one of the reported indicators and the three methods for estimating population proportions produced similar results (Table S1–S6 in File S1). However, given the interruption of the FSW study due to the political turmoil, the sample size was too small for *equilibrium* to be reached for some (11 of 54) indicators, including HIV status. Recruiter-recruit relationships were reciprocal with 95.6% of recruited MSM and 85.5% of recruited FSW peers indicating during screening that they would also have given a coupon to the same person who gave them their coupon.

During screening we also enquired about indicators for a potential overlap of networks in terms of being acquainted with a large number of persons from other most vulnerable groups. We found that 12% of the 227 MSM were acquainted with more than five PWID, and 13% of MSM knew more than five FSW. In turn, 17% of the 69 FSW in the sample indicated being acquainted with more than five MSM, and 13% of FSW knew more than five PWID.

Table 1 shows population estimates of sociodemographic characteristics for both groups.

**Table 1:** Socio-demographic characteristics and prevalence of HIV and other infections among MSM and FSW in Tripoli, Libya, 2010.

Indicators	Men having sex with men				Female sex workers			
		n*	%†	95% CI		n*	%†	95% CI
*Sociodemographic indicators*								
Age	E1							
15–19		70	31.9	(22.5, 41.9)		2	0.0	|
20–29		134	59.5	(50.2, 68.9)		35	73.4	|
30–39		14	5.5	(2.4, 9.2)		19	17.7	|
40–49		9	3.2	(0.7, 5.8)		10	8.9	|
≥ 50		0	0.0	(0, 0)		3	0.0	|
Civil status	E1				E1			
Married, living with spouse		1	0.0	(0, 0)		1	2.9	(0, 7)
Married, living with other sexual partner		0	0.0	(0, 0)		0	0.0	(0, 0)
Married, not living with spouse/ sexual partner		0	0.0	(0, 0)		4	4.6	(0.9, 10.3)
Not married, living with sexual partner		1	0.0	|		17	31.5	(17.4, 44.2)
Not married, not living with sexual partner		221	98.5	(95.4, 99.7)		47	61.1	(47.8, 76.7)
No response		4	1.5	(0.3, 4.6)		0	0.0	(0, 0)
Country of origin	E1				E1			
Libya		224	100.0	(100, 100)		8	12.4	(0, 29.4)
Abroad		3	0.0	|		61	87.6	(70.5, 100)
Time period lived in Libya	E1							
<10 years		4	1.5	(0, 5.7)		48	82.7	(62.6, 95.8)
10–20 years		73	33.7	(24.6, 43.4)		11	4.6	(0.7, 10.9)
>20 years		150	64.8	(54.7, 73.6)		10	12.7	(1, 30.8)
Travelled for work for >1 month during past year			−			8	20.5	(4.1, 49.6)
Education level ∧	E1							
Less than higher completed		97	45.1	(37.9, 53.4)		49	81.3	(69.5, 90.8)
Higher complete		80	32.5	(25.6, 39.4)		7	10.8	(3.4, 21.3)
Above higher		49	22.4	(16, 28.3)		7	5.9	(0.1, 13.2)
No response		1	0.0	(0, 0)		6	2.0	(0, 6.5)
Main source of income/ employment	E1				E1			
None		111	52.1	(43.5, 60.5)		42	66.4	(47.1, 82.8)
Household/domestic		0	0.0	(0, 0)		19	25.3	(11.9, 44.1)
Professional/businessman		6	2.1	(0.4, 4.3)		1	0.0	(0, 0)
Employee		16	5.4	(2.6, 8.3)		0	0.0	(0, 0)
Mechanic/factory worker/labourer		4	1.5	(0.2, 3.3)		0	0.0	(0, 0)
Hairdresser/salon worker/shop/sales/service		23	12.6	(7.6, 17.4)		7	8.3	(2.3, 13.6)
Taxi/bus/truck driver		6	2.5	(0.7, 4.8)		0	0.0	(0, 0)
Watchman/security guard		9	4.9	(1.6, 9.4)		0	0.0	(0, 0)
Hawker/street vendor/casual labourer		47	17.9	(13.2, 24)		0	0.0	(0, 0)
Other		5	1.0	(0.1, 2.4)		0	0.0	(0, 0)
*Biological indicators*								
HIV infection	E1							
Yes		12	3.1	(0.7, 6.9)		7	15.7	(3.2, 32.6)
No		215	96.9	(93.1, 99.3)		61	83.7	(66.7, 95.9)
Missing data ‡		0	0.0	(0,0)		1	0.6	(0, 2.6)
Hepatitis B infection	E1							
Yes		7	2.9	(0.9, 6.1)		2	0.0	|
No		217	95.8	(92.1, 98.2)		67	100.0	(100, 100)
Missing data‡		3	1.2	(0, 3.3)		0	0.0	(0, 0)
Hepatitis C infection	E1				E1			
Yes		19	7.3	(2.3, 13.7)		5	5.2	(1.2, 12.2)
No		205	91.4	(84.8, 96.4)		64	94.8	(87.8, 98.8)
Missing data‡		3	1.3	(0, 3.4)		0	0.0	(0, 0)
HIV/Hepatitis B co-infection	E1				E1			
Yes		0	0.0	(0,0)		0	0.0	(0, 0)
No		224	98.7	(96.5, 100)		68	99.3	(97, 100)
Missing data‡		3	1.3	(0, 3.5)		1	0.7	(0, 3)
HIV/Hepatitis C co-infection	E2				E1			
Yes		10	2.1	(0, 5.5)		3	3.7	(0, 12.6)
No		214	96.6	(92.7, 99.1)		65	95.6	(86.7, 100)
Missing data‡		3	1.3	(0, 3.4)		1	0.7	(0, 2.7)

* Sample size n out of total of N = 227 for MSM and N = 69 for FSW (seeds not included)

† Population estimates computed using RDSII/DA estimator method [30]

∧ Level of education is defined as follows: “less than higher” means 1–9 years of school, “higher” means 10–12 years of school, and “above higher” corresponds to undergraduate and postgraduate education.

‡ Data missing due to laboratory error

| Insufficient data to compute reliable 95% CI (Confidence Interval)

E1 = *Equilibrium* reached at 1% level, E2 = *Equilibrium* reached at 2% level

### HIV, HBV and HCV Prevalence

For MSM we estimated an HIV prevalence of 3.1%, HBV prevalence of 2.9%, and HCV prevalence of 7.3% (Table 1). None of the MSM were HIV/HBV co-infected, but 2.1% were HIV/HCV co-infected. Among FSW, the estimated HIV-prevalence amounted to 15.7%. The estimated RDS-adjusted HBV prevalence was 0% and there were no cases of HIV/HBV co-infection. In contrast, the HCV prevalence was 5.2%, with an HIV/HCV co-infection rate of 3.7%.

### Risk Behaviours

For MSM the median age of first anal intercourse with a man was 17 years. An estimated 58.1% of MSM had their first sexual intercourse with males before reaching 18 years, and 13.8% had a forced sexual debut (Table 2). 44.2% of respondents reported having had >3 anal sex partners during the past 6 months, and 16.9% had group sex at some point in the past. Only 21% of MSM used a condom during last anal intercourse. More than half of the respondents (54.3%) reported ever having used lubricants, of which only 0.6% were the recommended water-based lubricants. 26.5% of MSM had anal intercourse with a commercial partner in the past six months, but only 19.4% of these used a condom at last commercial intercourse.

**Table 2:** Sexual behaviour and risk factors for HIV infection among MSM in Tripoli, Libya, 2010.

Risk factors and other indicators		n*	(n^POS^;n^NEG^)	Proportion of participants		Association with HIV status		
				%†	95% CI	OR‡	95% CI	
Sexual behaviour with males								
Age at first sexual intercourse	E1							
<18 years		142	(1;141)	58.1	(48.7, 67.2)	Ref		
≥18 years		83	(11;72)	40.7	(31.5, 50.1)	21.3***	(3.0, 929.2)	
Don’t know		2	(0;2)	1.2	(0.0, 3.8)	0.0	(0.0, 2654.2)	
Forced sexual debut	E1							
No		192	(10;182)	85.1	(79.4, 90.7)	Ref		
Yes		33	(2;31)	13.8	(8.0, 19.1)	1.2	(0.1, 5.9)	
No response		2	(0;2)	1.16	(0.0, 3.3)			
Was forced to sexual intercourse in past 12 months	E1							
No		215	(11;204)	94.8	(90.9,97.3)	Ref		
Yes		12	(1;11)	5.2	(2.7,9.1)	1.7	(0.0, 13.81)	
Anal sex with multiple partners (>1) in past 6 months	E1							
No		19	(0;19)	12.3	(12.3, 7.3)	Ref		
Yes		207	(12;195)	87.5	(81.1, 92.4)	||		
Missing		1	(0;1)	0.2	(0.0, 1.0)	||		
Number of anal sex partners in past 6 months	E1							
≤3		108	(3;105)	55.8	(47.5, 63.9)	Ref		
>3		119	(9;110)	44.2	(36.1, 52.5)	2.9	(0.7, 16.8)	
Ever insertive anal sex	E1							
No		3	(0;3)	0	(0.0, 0.0)	Ref		
Yes		223	(12;211)	98.0	|	||		
Missing		1	(0;1)	2	(0.0, 1.7)	||		
Insertive anal sex during past 6 months	E1							
No		2	(0;2)	0,9	(0.0, 2.6)	Ref		
Yes		220	(12;208)	97.6	(94.7, 99.0)	||		
Missing		5	(0;5)	1.5	(0.4, 3.9)	||		
Number of insertive partners	E1							
1		23/220	(0;23)	13.7	(8.4,19.5)	Ref		
2 to 4		124/220	(9;115)	56.4	(49.2,64.8)	||		
>4		73/220	(3;70)	29.9	(21.7,37.3)	||		
Ever receptive anal sex								
No		205	(11;194)	93.7	(88.0, 97.9)	Ref		
Yes		22	(1;21)	6.3	(2.1, 11.7)	0.84	(0.0, 6.3)	
Receptive anal sex during past 6 months	E2							
No		5	(0;5)	2.7	(0.6, 6.0)	Ref		
Yes		17	(1;16)	3.8	(1.1, 7.7)	||		
Number of partners	E2							
1		2/17	(0;2)	0.0	|	Ref		
2 to 4		3/17	(0;3)	38.9	(0.0, 91.1)	||		
>4		11/17	(1;10)	56.6	(4.0, 100.0)	||		
Don’t know		1/17	(0;1)	4.5	(0.0, 17.8)	||		
Ever had group sex	E1							
No		185	(11;174)	83.1	(77.1, 89.0)	Ref		
Yes		42	(1;41)	16.9	(10.9, 22.9)	0.4	(0.0, 2.8)	
Had group sex during past 6 months	E2							
No		205	(11;194)	91.2	(86.7, 94.9)	Ref		
Yes		22	(1;21)	8.8	(5.1, 13.3)	0.8	(0.0, 6.3)	
Number of partners								
2 to 5	E1	19/22	(1;18)	87.2	(66.2, 100.0)	Ref		
6 to 8		3/22	(0;3)	12.8	(0.0, 33.8)	0.0	(0.0, 246.1)	
Condom use during last anal sex	E1							
No		172	(5;167)	79.0	(72.1, 85.3)	Ref		
Yes		55	(7;48)	21.0	(14.5, 27.7)	4.8	(1.3, 20.2)	
Condom break during anal sex in last month	E1							
No		214	(5;70)	94.5	(91.6, 97.6)	Ref		
Yes		13	(1;12)	5.5	(2.4, 8.4)	0.2	(0.0, 0.8)	
Ever used lubricants	E1							
No		100	(6;94)	45.7	(38.1, 54)	Ref		
Yes		127	(6;121)	54.3	(45.8, 61.8)	0.8	(0.2, 3.0)	
Type of lubricants commonly used								
Oil based	E1	46/127	(4;42)	39.3	(30.3, 50.7)	Ref		
Water based	E1	1/127	(0;1)	0.6	(0.0, 2.8)	0.0	(0.0, 416.5)	
No response	E1	80/127	(2;78)	60.1	(48.5, 69.2)	0.3	(0.0, 2.0)	
Consistent lubricant use during past 6 month	E1							
No		197	(10;187)	88.7	(83.4, 93.7)	Ref		
Yes		30	(2;28)	11.3	(6.2, 16.5)	1.6	(0.1, 12.1)	
Had anal sexual intercourse with regular non-commercial partner in past 6 months	E1							
No		40	(1;39)	20.9	(14.2, 29.0)	Ref		
Yes		187	(11;176)	79.2	(71.3, 85.7)	2.4	(0.3, 107.5)	
Used a condom at last sex	E1							
No		138/187	(6;132)	77.4	(14.7, 30.3)	Ref		
Yes		48/187	(5;43)	21.8	(14.4, 30)	2.5	(0.6, 10.6)	
Don’t remember		1/187	(0;1)	0.5	(0.0, 2.0)	0.0	(0.0, 853.0)	
Consistent condom use	E1							
No		164/187	(8;156)	89.6	(84.0, 94.4)	Ref		
Yes		23/187	(3;20)	10.4	(5.6, 15.8)	2.9	(0.5, 13.4)	
Had anal sexual intercourse with non-regular non-commercial partner in past 6 months	E1							
No		71	(4;67)	31.0	(23.2, 38.3)	Ref		
Yes		156	(8;148)	69.0	(61.8, 76.7)	0.9	(0.2, 4.3)	
Used a condom at last sex	E1							
No		118/156	(6;112)	79.5	(71.8, 86.3)	Ref		
Yes		38/156	(2;36)	20.5	(13.4, 28.2)	1.0	(0.0, 6.1)	
Consistent condom use	E1							
No		128/156	(6;122)	84.2	(76.6, 90.1)	Ref		
Yes		28/156	(2;26)	15.8	(9.8, 23.6)	1.6	(0.1, 9.4)	
Had anal sexual intercourse with commercial partner in past 6 months	E1							
No		158	(8;150)	73.5	(66.1, 80.6)	Ref		
Yes		69	(4;65)	26.5	(19.3, 33.9)	1.2	(0.2, 4.5)	
Used a condom at last sex	E1							
No		49/69	(3;46)	80.6	(67.5, 89.8)	Ref		
Yes		20/69	(1;19)	19.4	(10.1, 32.2)	0.8	(0.0, 10.8)	
Consistent condom use	E1							
No		56/69	(3;53)	86.3	(74.4, 94.0)	Ref		
Yes		13/69	(1;12)	13.7	(6.0, 25.6)	1.5	(0.0, 20.1)	
Had oral sex (with any partner) during past 6 months	E1							
No		152	(8;144)	69.6	(62.1,76.9)	Ref		
Yes		75	(4;71)	30.4	(23.1,37.9)	1.0	(0.2, 4.0)	
Used a condom at last sex	E1							
No		63/75	(3;60)	88.1	(78.9, 94.7)	Ref		
Yes		12/75	(1;11)	11.9	(5.2, 21.3)	1.8	(0.0, 24.9)	
Consistent condom use	E1							
No		67/75	(3;64)	92.8	(86.6, 97.7)	Ref		
Yes		8/75	(1;7)	7.2	(2.3, 13.5)	3.0	(0.1, 43.6)	
Ejaculated in partner’s mouth or partner ejaculated in his mouth	E1							
No		42/75	(1;41)	54.2	(41.2, 67.7)	Ref		
Yes		33/75	(3;30)	45.2	(32.1, 58.9)	4.0	(0.3, 220.1)	
Sexual behaviour with females								
Ever had sex with female partner	E1							
No		68	(4;64)	31.5	(23.0, 40.2)	Ref		
Yes		159	(8;151)	68.5	(59.7, 77.0)	0.8	(0.2, 4.0)	
Age at first sexual intercourse								
<18	E1	76/159	(1;75)	44.3	(34.0, 54.9)	Ref		
≥18		77/159	(6;71)	51.8	(41.4, 61.4)	6.3	(0.7, 294.9)	
No response		6/159	(1;5)	4.0	(1.2, 7.7)	13.8	(0.2, 1177.8)	
Had sex with female partner in last six months	E1							
No		33	(0;33)	16.1	(10.6,22.1)	Ref		
Yes		125	(8;117)	53.0	(44.4,61.1)	||		
Missing		1	(0;1)	0.4	(0.0, 1.8)	||		
Never had sex with females		68	(4;64)	30.5	(21.9, 39.6)	||		
Had their first sexual intercourse with**	E1							
a woman		35	(3;32)	14.1	(9.5,19.5)	Ref		
a man		96	(4;92)	41.4	(33.7,48.5)	0.5	(0.1, 3.4)	
Cannot be determined (same reported age at first sex)		21	(0;21)	11.4	(6.0,18.2)	0.0	(0.0, 4.0)	
Cannot be determined (missing reported age at first sex)		7	(1;6)	3.4	(1.0,6.6)	1.8	(0.0, 26.6)	
Not applicable (never had sex with a woman)		68	(4;64)	29.7	(20.9,38.5)	0.7	(0.1, 4.8)	
Men who have risky sex with men and women††	E1							
No		134	(7;127)	61.5	(53.5, 69.2)	Ref		
Yes		93	(5;88)	38.5	(30.6, 46.5)	1.0	(0.2, 3.9)	
Other risk factors								
Consumed alcohol ≥ 4 times per week in past 6 months	E1							
No		220	(12;208)	96.3	(93.2, 98.6)	Ref		
Yes		7	(0;7)	3.7	(1.4, 6.8)	0.0	(0.0, 13.5)	
Non-injecting drug use in past 6 months	E1							
No		150	(0;150)	70.5	(61.8,78.8)	Ref		
Yes		77	(12;65)	29.5	(21.1, 38.2)	||		
Ever injected drugs	E1							
No		216	(2;214)	96.0	(91.5, 99.0)	Ref		
Yes		11	(10;1)	4.0	(1.0, 8.6)	802.0***	(72.7, 4.5e15)	
Age of first injection	E1							
> = 21		10/11	(9;1)	100.0	(100.0, 100.0)	Ref		
<21		1/11	(1;0)	0.0	(0.0, 0.0)	0.0	(0.0, 388.0)	
Frequency of injection drug use during past 6 months	E1							
≤ once per week		1/11	(1;0)	7.2	(0.0, 43.1)	Ref		
> once per week		10/11	(9;1)	92.8	(55.9, 100.0)	||		
Shared needle/syringe at last injection	E1							
No		9/11	(8;1)	100.0	|	Ref		
Yes		2/11	(2;0)	0.0	|	||		
Perception of risk for HIV infection	E1							
High risk		17	(9;8)	5.7	(3.2, 9.7)	115.4***	(13.5, 5387.0)	
Medium risk		42	(1;41)	19.5	(13.9, 26.1)	2.7	(0.0, 217.8)	
Low risk		33	(1;32)	15.4	(10.2, 20.8)	3.5	(0.0, 278.9)	
No risk		114	(1;113)	49.8	(41.1, 57.1)	Ref		
Don’t know		21	(0;21)	9.6	(5.6, 14.4)	0.0	(0.0, 211)	

* Sample size n out of total of N = 227 where not indicated otherwise (seeds not included)

† Population estimates computed using RDSII/DA estimator method [30]

‡ OR: Odds Ratio showing association between the characteristic and testing HIV positive (based on Fisher’s exact method).

| Insufficient data to compute reliable 95% CI (Confidence Interval)

** Variable was derived from reported ages at first intercourse with a man and a woman

†† Respondents who had unprotected sex with a woman at least once in the last 6 months, and who have had unprotected anal sex with at least one other man in the last 6 months

|| Odds ratio could not be computed due to zero cell count(s)

*** p-value <0.001

n^POS^, n^NEG^ = Number of HIV positive and negative participants for this row

E1 = *Equilibrium* reached at 1% level, E2 = *Equilibrium* reached at 2% level

More than two thirds (68.5%) of MSM also had sexual intercourse with a female partner in the past, and more than half (53%) had intercourse with a woman in the last six months. The median reported age of first intercourse with a woman was 18 years. 38.5% of MSM reported risky sex with both men and women during the past six months. Almost one third (29.5%) of respondents had used non-injecting drugs in the past six months, such as Hashish or Tranquilizers, and 4% reported having ever injected drugs. Only 5.7% of MSM felt they were at high risk for HIV infection.

Table 3 provides information about FSW commercial sex history. 18.2% of FSW reported having been forced to have sex during the past year. 42.8% of FSW had 10–50 sex partners during the past six months and 30.5% had 50 or more. 63.4% of FSW reported consistent condom use during sex with one-time clients, and 56.8% during sex with regular clients. 27.8% of FSW experienced STI symptoms in the past year. Only two respondents had ever injected drugs.

**Table 3:** Sexual behaviour and risk factors for HIV infection among FSW in Tripoli, Libya, 2010.

Risk factors and other indicators		n*	(n^POS^;n^NEG^)	Proportion of participants	
				%†	95% CI
Age at first sexual intercourse	E1				
<18		20	(2;18)	22.7	(10.1, 37)
≥18		48	(5;42)	75.5	(58.9, 89.1)
No response		1	(0;1)	1.8	(0, 7.3)
Age at first selling sex					
<18		5	(0;5)	2.4	(0, 7.9)
≥18		63	(7;55)	96.2	(89.7, 99.6)
no response		1	(0;1)	1.3	(0, 5.3)
Was forced to sexual intercourse in past 12 months	E1	13	(1;12)	18.2	(8.4, 33.4)
Reasons for exchanging money or goods for sex	E1				
economic		58	(4;54)	90.1	(80.1, 96.4)
forced		4	(0;4)	3.8	(0.8, 7.8)
abandoned by family/husband		6	(2;3)	6.1	(1.2, 14.4)
other		1	(1;0)	0.0	(0, 0)
Main place where respondent met clients during past month					
Own home		14	(0;14)	14.6	(0.9, 32.3)
Friend’s home		9	(0;9)	10.8	(4, 25)
Sex Partner’s home		10	(0;10)	11.3	(2.5, 22.4)
On the streets		8	(2;5)	20.0	(4, 36.8)
By telephone		5	(1;4)	6.0	(0.6, 12.1)
In Café		8	(2;6)	15.6	(2.9, 36.2)
Brothel/ Connection house		6	(0;6)	8.6	(0, 23.9)
Other		6	(2;4)	10.2	(1.7, 18)
No response		2	(0;2)	2.1	(0, 7.6)
Missing data		1	(0;1)	0.8	(0, 4.1)
Total number of sexual partners in last six months					
<10		26	(2;23)	25.9	(15, 40.6)
10 to 50		23	(4;19)	42.8	(24.8, 57.8)
≥50		17	(1;16)	30.5	(16.1, 47.6)
Don’t know		3	(0;3)	0.9	(0, 3.2)
Number of regular clients in last six months					
<10		9	(1;8)	22.6	(5.7, 36.7)
10 to 50		12	(1;11)	10.8	(1.8, 24.6)
≥50		18	(0;18)	21.4	(12.5, 34.8)
Don’t know		30	(5;25)	45.2	(30.6, 62.5)
Number of one-time clients in last six months	E1				
<10		56	(6;49)	85.2	(73.2, 90.3)
10 to 50		6	(1;5)	8.3	(3.2, 17.7)
≥50		3	(0;3)	3.6	(0, 10.9)
Don’t know		4	(0;4)	2.9	(0, 7.6)
Had sex with one-time client	E1	36	(6;30)	51.8	(37.9, 69.3)
Used condom consistently	E1	24/36	(6;18)	63.4	(41.4, 81.7)
Used a condom at last sex	E1	29/36	(6;23)	83.1	(66.5, 95.4)
Reasons for not using a condom at last sex ‡	E1				
Not available		1/36	(0;1)	1.1	(0, 4.8)
Not pleasurable for client		1/36	(0;1)	0.0	(0, 0)
Didn’t think of it		1/36	(0;1)	0.0	(0, 0)
Condom negotiation at last sex with one-time client	E1				
Respondent suggested condom use		26/36	(6;20)	54.2	(31.3, 81.8)
Client suggested condom use		3/36	(0;3)	17.3	(4.3, 31.4)
Joint decision		1/36	(0;1)	11.6	(0, 33.3)
Not applicable/no response		6/36	(0;6)	16.9	(3.7, 32.3)
Had sex with regular client	E1	38	(1;36)	54.3	(37.5, 70.3)
Used condom consistently	E1	22/38	(1;20)	56.8	(39.2, 74.3)
Used a condom at last sex	E1	27/38	(1;25)	76.7	(61.1, 88.7)
Reasons for not using a condom with last regular client ‡	E1				
Not available		2/38	(0;2)	3.3	(0, 10.2)
Not pleasurable for respondent		2/38	(0;8)	2.7	(0, 8.2)
Not pleasurable for client		8/38	(0;1)	5.1	(2, 11.6)
Didn’t think of it		1/38	(0;4)	0.0	(0, 0)
Trust partner		4/38	(0;1)	3.0	(0.6, 9.4)
No response		1/38	(0;2)	0.0	(0, 0)
Condom negotiation at last sex with regular client	E1				
Respondent suggested condom use		24/38	(0;23)	71.8	(46.7, 78.8)
Client suggested condom use		2/38	(1;1)	0.0	|
Joint decision		1/38	(0;1)	3.9	(0, 11.9)
No response		11/38	(0;11)	24.3	(18.1, 48.7)
Ever had anal sex	E1	2	(0;2)	1.5	(0, 4.4)
Had anal sex in last 30 days and used condom at last sex	E1	0/1	(0;0)	0	(0, 0)
Ever had dry sex	E1	19	(0;19)	33.9	(17.3, 48.8)
Had dry sex in last 30 days and used condom at last dry sex	E1	8/12	(0;8)	67.4	(29.1, 94.6)
Reported STI symptom (unusual genital discharge, ulcer or sore) during last year	E2	21	(1;20)	27.8	(15, 42.2)
Correct action taken for STI **	E2	7/21	(0;7)	24.3	(8.8, 51.2)
Consumed alcohol ≥ 4 times per week in past 6 months	E1	3	(1;2)	0.0	|
Non-injecting drug use in past 6 months	E1	1	(1;0)	1.2	(0, 4.3)
Ever injected drugs		2	(2;0)	0.0	|

* Sample size n out of total of N = 69 where not indicated otherwise (seeds not included)

† Population estimates computed using RDSII/DA estimator method [30]

‡ Multiple answers possible

| Insufficient data to compute reliable 95% CI (Confidence Interval)

** Took at least two of the following actions: sought care at public health facility, sought care at private health facility, told sex partner about symptoms, stopped having sex, used condom during sex

n^POS^, n^NEG^ = Number of HIV positive and negative participants for this row (Note: one participant is missing HIV status)

E1 = *Equilibrium* reached at 1% level, E2 = *Equilibrium* reached at 2% level

### Access to Services, Knowledge and Attitudes

Access to HIV prevention services and HIV-related knowledge was limited (Table 4). Only 0.9% of MSM and 0% of FSW have been exposed to prevention programmes related to both HIV testing and condoms. Although most MSM (94.6%) and FSW (81.7%) knew how to obtain condoms, 0% of MSM and 1.1% of FSW had a condom to hand on the survey day, and only 12.1% of MSM and 49.2% of FSW knew how to use condoms correctly. 45.6% of MSM and 38.6% of FSW had been tested for HIV during the past year and knew the result. Only 16.8% of MSM and 18.6% of FSW succeeded in both, correctly identifying ways to prevent sexual HIV transmission and rejecting major misconceptions about HIV transmission. STI-related knowledge was very poor with only 1.3% of MSM and 5.4% of FSW correctly identifying at least two common STI symptoms in both men and women. Only 13.1% of MSM and 26.2% of FSW reported no stigmatizing attitudes against people living with HIV (PLHIV).

**Table 4:** Access to services, knowledge and attitudes related to HIV among MSM and FSW in Tripoli, Libya, 2010.

Indicators	Men having sex with men				Female sex workers			
		n*	%†	95% CI		n*	%†	95% CI
Exposure to HIV prevention programmes ‡	E1	3	0.9	(0, 2.3)	E1	0	0.0	(0, 0)
Condom use								
Respondents with condoms to hand |	E1	1	0.0	(0, 0)	E1	2	1.1	(0, 3.5)
Knowledge of correct condom use	E1	31	12.1	(7.4, 16.5)	E1	34	49.2	(34.3, 64.6)
Knowledge of how to obtain condoms	E1	214	94.6	(91.2, 97.2)	E1	57	81.7	(69, 90.2)
Knowledge of place to get female condoms		−				9	8.2	(1.2, 17.9)
Knowledge of where to get lubricants	E1	177	75.7	(69.7, 81.9)	−			
Underwent an HIV test in past 12 months, and knows results	E1	106	45.6	(38.7, 53.5)	E1	32	38.6	(25.1, 55.3)
HIV-related knowledge								
Correctly identified ways to prevent sexual transmission of HIV and who reject major misconceptions about HIV transmission**	E1	42	16.8	(11.6, 22.7)	E1	16	18.6	(10.1, 34.9)
Knowledge of mother to child transmission		−			E1	4	3.3	(0.6, 8.1)
STI-related knowledge: Correctly identified at least two common signs/symptoms of STIs in both men and women	E1	3	1.3	(0, 3.5)	E1	7	5.4	(1.5, 14.6)
Has been arrested during past 12 months	E1	23	10.9	(6.4, 16.9)	E1	3	5.9	(0, 12.1)
Main reason for arrest	E1				E1			
Being on/ possession of drugs		2	4.6	(0, 15.5)		0	0.0	(0, 0)
Being drunk		4	13.4	(0, 32.1)		0	0.0	(0, 0)
Fight		10	46.2	(21.1, 73.9)		0	0.0	(0, 0)
Exchanging sex for money		0	0.0	(0, 0)		1	0.0	(0, 0)
Other		6	35.8	(9, 59.8)		1	0.0	(0, 0)
No response		1	0.0	(0, 0)		0	0.0	(0, 0)
Stigma & discrimination								
Absence of stigma towards people living with HIV ††	E1	31	13.1	(8.8, 19.5)		16	26.2	(11.8, 41.9)
Has been refused different services in the last 12 months because (s)he is believed to have sex with men/ exchange sex for money or goods ‡‡	E1	12	5.2	(1.9, 10.3)	E1	9	9.8	(2.9, 18.6)
At least one verbal insult experienced by respondent in the last 12 months because he is believed to be MSM/ FSW	E1	24	9.1	(5.6, 13.9)	E1	22	28.3	(15.8, 40.5)
Has been hit, kicked, or beaten in the last 12 months because (s)he is believed to have sex with men/ exchange sex for money or goods	E1	2	0.8	(0, 2.2)	E1	6	11.0	(3.4, 20.6)

* Sample size n out of total of N = 227 for MSM and N = 69 for FSW (seeds not included)

† Population estimates computed using RDSII/DA estimator method [30]

‡ Respondents who know where they can get tested for HIV and who have been given condoms through outreach service, drop-in centre or health facility in past 12 months

| Respondents who could show at least one condom to interviewer

** Respondents who know that a healthy-looking person can transmit HIV, that the transmission risk can be reduced by having sex with only one faithful, uninfected partner, and by using condoms, and who reject the misconceptions that HIV can be transmitted by sharing a meal with someone infected and through mosquito bites.

†† Respondents who would be willing to share a meal with a person who has HIV or AIDS, would be willing to care at their house for a male or female relative who is ill with HIV, who would buy food from a shopkeeper or food seller who has HIV, who thinks a student infected with HIV, but is not sick with AIDS should be allowed to attend school, and a teacher who is infected with HIV, but is not sick should be allowed to continue to teach.

‡‡ Respondents who have been refused health care, employment, education, restaurant service or police assistance

E1 = *Equilibrium* reached at 1% level, E2 = *Equilibrium* reached at 2% level

### Factors Associated with HIV Seropositivity among MSM

Using Fisher’s central exact test we identified three factors associated with HIV sero-positivity among MSM (Table 2). The odds of HIV infection were 802 times higher among individuals who ever injected drugs, but the confidence interval was very wide (CI 72.7, 4.5e15). This is because some of the numbers were small, with only 1 of the 11 MSM who ever injected drugs testing HIV negative, and only 2 of the 12 HIV positive MSM in the sample indicating that they had never injected drugs. Odds ratios (OR) for associations with HIV sero-positivity could not be computed for the factors “needle sharing at last injection” and “non-injecting drug use in the past six months”, because of “zero cells”, i.e. none of those MSM who shared needles at last injection were HIV negative, and none of those who had never used non-injecting drugs were HIV positive. In addition, MSM who had been at least 18 years old at their first sexual intercourse were more likely to be HIV positive (OR 21.3, CI 3.0, 929.0), but this factor might be confounded by the fact that none of those MSM who had sex before the age of 18 had ever injected drugs. Further, those MSM who perceived themselves at high risk of HIV infection were more likely to be HIV positive (OR 115.0, CI 13.5, 5387.0).

Numbers were too small to fit a model that adjusts for all potential confounders, but the following additional univariate correlations (not shown in table) might assist in the interpretation of some of the results. Respondents who had ever injected drugs were more likely to perceive being at high or medium risk of HIV infection (OR 29.3, CI 4.0, 1292.6), more likely to have taken an HIV test and obtained their result in the 12 months preceding the survey (OR 12.4, CI 1.7, 545.5) and more likely to report using a condom during their last anal sex (OR 4.1, CI 1.0, 17.6) as compared to non-injectors. MSM who used a condom during last anal sex were also more likely to have taken an HIV test in the past year and obtained the result (OR 2.5, CI 1.3, 4.9). MSM who were ≥35 years old were more likely to be HIV positive (OR 82.2, CI 16.4, 570.5), but were also more likely to have ever injected drugs (OR 309.0, CI 35.6, 13424.2).

## Discussion

An HIV prevalence of 3.1% among MSM in Tripoli indicates an HIV epidemic that is approaching the 5%-threshold of a concentrated epidemic [14]. Although the HIV prevalence among FSW is 15.7%, no definite classification is possible due to the small sample size. It is possible that the HIV epidemic among FSW in Libya is already or will soon become concentrated. So far, only a concentrated epidemic among PWID had been confirmed by our recent study in Libya [13].

There are no previous studies among MSM and FSW in Libya, nor studies among the general population stratified for sexually active males and females, to which our data could be compared [3], [12]. However, looking at recent reports from other North-African countries, the HIV prevalence among MSM in our study seems comparably slightly lower [12], [31], [32], [33], [34] and the HIV prevalence estimate among FSW comparably higher [31], [32], [33], [35]. However, the prevalence varies for different cities in these countries, and more details on the age distributions would be needed for more informative comparisons as age is often associated with HIV-status.

Our study further detected a relatively moderate HBV prevalence of 2.9% among MSM and a 0% among FSW. In contrast, HCV prevalence was relatively high among MSM (7.3%) and FSW (5.2%). It is interesting that our study did not show any cases of HIV/HBV co-infection, but revealed HIV/HCV co-infection of 2.1% for MSM and 3.7% for FSW. This pattern might point to parenteral routes of HIV transmission given that HCV (in comparison to HBV) is more easily transmitted via unsafe injections than by mucosal exposure during sexual intercourse [36]. However, these results should be interpreted with caution since the confidence intervals include the value zero, indicating the eventuality of no existing co-infection.

Sexual risk factors among MSM included a high number of anal sex partners and low use of condoms and the recommended water-based lubricants. In addition, a substantial proportion of MSM had group sex and commercial sex. Sexual risk factors among FSW included a high number of sex partners, and a condom use rate that was higher than among MSM, but still insufficient to ensure consistent protection. This may explain why a substantial proportion of FSW experienced STI symptoms during the past year. Not only a high rate of partner change, and paid sex, but also a history of STIs have been identified as key risk factors of HIV transmission in both early and advanced epidemics [37].

Additional risk factors in our study include MSM taking illicit non-injecting drugs. People who use non-injecting drugs have recently been identified as an overlooked population with a substantial burden of HIV due to their high-risk behaviours and overlapping social and sexual networks with PWID [38]. Although only 4% of MSM and two FSW in our study reported ever having injected drugs, IDU is likely to more or less directly fuel the spread of HIV among MSM and FSW. Both FSW and 10 out of the 11 MSM who admitted having used injecting drugs were HIV positive.

Sample size restrictions did not allow us to run reliable correlation analyses among FSW, but the analyses among MSM showed that IDU was the highest risk factor for HIV transmission, although due to small cell counts the confidence interval is very wide and the result has to be interpreted with caution. Given the recently detected HIV prevalence of 87% among PWID in Tripoli [13], there is a risk for an expansion of the epidemic not only to and within different most vulnerable populations (MVPs), but also to the general population via heterosexual transmission to clients and partners of FSW, PWID and MSM.

There are a few possible explanations for the relatively low prevalence of HIV among MSM detected in our study despite the high level of sexual risk behaviours and the lack of HIV-related knowledge. While the HIV epidemic is not new among PWID, who have one of the highest HIV prevalence rates in the world [13], it is most likely new among MSM. Furthermore, although a majority of HIV positive respondents reported ever injecting drugs overall there was only a small number of MSM who indicated having ever injected drugs (only 4 %). It is also very likely that the networks of MSM who inject drugs are isolated from the networks of heterosexual drug injectors due to stigma. This, however, does not mean that the situation is stable and could not change. The HIV epidemic among PWID may penetrate MSM networks at any time, leading to a sharp increase in the prevalence of HIV among MSM. Moreover, given that only a fifth of respondents reported having used a condom at last sex, and given that close to 40% of MSM had unprotected sex with both men and women in this time period, the epidemic may quickly transfer to other population groups. An overlap and bridging between different MVPs has been described elsewhere in the Middle East and North Africa (MENA) [9], [12]. For example, in Iran and Pakistan, newly emerging epidemics among MSM have been linked to well established epidemics among PWID [9], [39].

A further risk factor for an expansion of the epidemic is the criminalization of FSW and MSM that pose profound structural barriers to HIV prevention and care in the MENA region [9], [40]. It drives the neediest populations away from services, increases their fear of disclosing risks to partners and providers, and renders them vulnerable to blackmail and abuse [41]. In Libya, sex work can result in a penalty of up to five years imprisonment for sex worker and client, and corporal punishment (100 lashes by flogging) of the sex worker. Legislation can apply the same law to punish MSM with imprisonment [11].

Fear of stigma and persecution was also a reason for the relatively slow recruitment of FSW. Slow FSW recruitment has also been reported elsewhere [19], [42], [43]. Given the outbreak of political turmoil in Libya, the FSW study prematurely ended and the target sample size could not be met. Hence, the FSW results, especially for those indicators, for which *equilibrium* could not be reached, have to be interpreted with caution, as they may not be representative for the whole FSW population.

Another study limitation is that, as in all cross-sectional studies, the relationships between cause and effect cannot be determined. Further, given that behavioural data were self-reported, social desirability bias might have led to over-reporting of preventive behaviour, such as condom use, and under-reporting of certain risk factors, such as non-injecting and injecting drug use, or being the receptive rather than the insertive sex partner. While 98.0% of MSM reported having insertive anal sex in the six months preceding the survey, only 6.3% admitted practicing receptive anal sex. Our preliminary social mapping of MSM revealed that practicing receptive sex was perceived as particularly stigmatizing among peers. Hence, it is possible that respondents tended to give a culturally more acceptable answer, claiming they practiced only insertive anal sex. Given that trafficking is frequent in the region, and that many FSW fear disclosing forced sex [40], it is also likely that some FSW (e.g. those held in connection houses or who met clients at their sex partner’s house) might have underreported forced sex as a reason for engaging in sex work. New studies are needed to explore the different types of FSW and MSM sub-groups in Libya, with results disaggregated for groups that might be particularly vulnerable to HIV, such as transgender people [2].

Despite the challenges and limitations described above, we showed that it is possible to engage MSM and FSW in Libya under difficult (pre-revolutionary) conditions and in the absence of pre-established links with hidden MVPs via NGOs. The results of the study show that an immediate response is crucial, in order to prevent the epidemic from spreading within different risk groups and to the general population. The risk of transmission is further fuelled by the recent political military events that led to increased migration, sexual and gender-based violence, the disruption of infection control and blood safety systems, and a nation-wide stock-out of antiretroviral drugs [3].

Comprehensive programmes for the prevention, treatment and care of HIV/AIDS and other STIs need to be rapidly and simultaneously scaled up among all MVPs and take account of the potential overlap of high-risk sexual and drug-using networks. Following international recommendations [2], [10], [40] legal and policy barriers to the scale-up of effective programmes should be removed, and attempts made to establish meaningful collaborations with newly founded civil society groups representing MVPs and PLHIV. Thereby, experiences from other MENA countries could be taken into account. For example, Djibouti has a history of protecting the rights of PLHIV, Iran has led in the development of a harm-reduction programme, and NGOs in Morocco have successfully worked with MSM and FSW for HIV and STI prevention [40].

A likely escalation of the epidemic in Libya can be averted if vigilant surveillance and evidence- and human-rights based programmes are implemented while the window of opportunity to act is still open [1].

## Supporting Information

File S1 Table S1 – Socio-demographic characteristics and prevalence of HIV and other infections among MSM in Tripoli, Libya, 2010. Table S2 – Socio-demographic characteristics and prevalence of HIV and other infections among FSW in Tripoli, Libya, 2010. Table S3 – Sexual behaviour and risk factors for HIV infection among MSM in Tripoli, Libya, 2010. Table S4 – Sexual behaviour and risk factors for HIV infection among FSW in Tripoli, Libya, 2010. Table S5 – Access to services, knowledge and attitudes related to HIV among MSM in Tripoli, Libya, 2010. Table S6 – Access to services, knowledge and attitudes related to HIV among FSW in Tripoli, Libya, 2010.
(DOC)
