# Supplementary material for: Filling the Knowledge Gap: Measuring HIV Prevalence and Risk Factors among Men Who Have Sex with Men and Female Sex Workers in Tripoli, Libya
Source: PLoS One. 2013 Jun 19;8(6):e66701. doi: 10.1371/journal.pone.0066701 (PMC3686727; doi:10.1371/journal.pone.0066701)
Supplement: File S1 — Table S1 – Socio-demographic characteristics and prevalence of HIV and other infections among MSM in Tripoli, Libya, 2010. Table S2 – Socio-demographic characteristics and prevalence of HIV and other infections among FSW in Tripoli, Libya, 2010. Table S3 – Sexual behaviour and risk factors for HIV infection among MSM in Tripoli, Libya, 2010. Table S4 – Sexual behaviour and risk factors for HIV infection among FSW in Tripoli, Libya, 2010. Table S5 – Access to services, knowledge and attitudes related to HIV among MSM in Tripoli, Libya, 2010. Table S6 – Access to services, knowledge and attitudes related to HIV among FSW in Tripoli, Libya, 2010. (DOC) [file pone.0066701.s001.doc]

Filling the knowledge gap: Measuring HIV prevalence and risk factors among men who have sex with men and female sex workers in Tripoli, Libya

**Supporting Information – Supplementary Result Tables**

Table S1 – Socio-demographic characteristics and prevalence of HIV and other infections among MSM in Tripoli, Libya, 2010

| Sociodemographic indicators | n* | RDSII/DA estimate† | |  | RDS I/DS estimate‡ | |  | RDS II estimate^ | |  |
| --- | --- | --- | --- | --- | --- | --- | --- | --- | --- | --- |
|  |  | % | 95% CI |  | % | 95% CI |  | % | 95% CI |  |
| Age |  |  |  |  |  |  |  |  |  | E1 |
| 15-19 | 70 | 31.9 | ( 22.5, 41.9) |  | 29.6 | ( 21.2, 38.5) |  | 31.7 | ( 22.4, 41.8) |  |
| 20-29 | 134 | 59.5 | ( 50.2, 68.9) |  | 61.2 | ( 52.6, 69.4) |  | 58.6 | ( 50.3, 68.9) |  |
| 30-39 | 14 | 5.5 | ( 2.4, 9.2) |  | 6.3 | ( 3, 10.1) |  | 6.2 | ( 2.4, 9.2) |  |
| 40-49 | 9 | 3.2 | ( 0.7, 5.8) |  | 2.9 | ( 0.7, 5.8) |  | 3.5 | ( 0.8, 5.8) |  |
| ≥ 50 | 0 | 0.0 | ( 0, 0) |  | 0.0 | ( 0, 0) |  | 0.0 | ( 0, 0) |  |
| Civil status |  |  |  |  |  |  |  |  |  | E1 |
| Married, living with spouse | 1 | 0.0 | ( 0, 0) |  | 0.0 | ( 0, 0) |  | 0.7 | | |  |
| Married, living with other sexual partner | 0 | 0.0 | ( 0, 0) |  | 0.0 | ( 0, 0) |  | 0.0 | ( 0, 0) |  |
| Married, not living with spouse/other sexual  partner | 0 | 0.0 | ( 0, 0) |  | 0.0 | ( 0, 0) |  | 0.0 | ( 0, 0) |  |
| Not married, living with sexual partner | 1 | 0.0 | ( 0, 0) |  | 0.0 | ( 0, 0) |  | 0.4 | | |  |
| Not married, not living with sexual partner | 221 | 98.5 | ( 95.4, 99.7) |  | 98.5 | ( 0, 99.3) |  | 97.5 | ( 94.8, 99.6) |  |
| No response | 4 | 1.5 | ( 0.3, 4.6) |  | 1.5 | ( 0.4, 99.6) |  | 1.5 | ( 0.3, 4.6) |  |
| Country of origin |  |  |  |  |  |  |  |  |  | E2 |
| Libya | 224 | 100.0 | ( 100, 100) |  | 100.0 | ( 100, 100) |  | 100.0 | ( 100, 100) |  |
| Abroad | 3 | 0.0 | | |  | 0.0 | | |  | 0.7 | | |  |
| Time period lived in Libya |  |  |  |  |  |  |  |  |  | E1 |
| <10 years | 4 | 1.5 | ( 0, 5.7) |  | 1.9 | ( 0, 6.3) |  | 1.0 | ( 0, 6) |  |
| 10-20 years | 73 | 33.7 | ( 24.6, 43.4) |  | 33.0 | ( 0, 41.5) |  | 34.3 | ( 24.7, 43.3) |  |
| >20 years | 150 | 64.8 | ( 54.7, 73.6) |  | 65.1 | ( 30.3, 73.3) |  | 64.7 | ( 54.8, 73.5) |  |
| Education level |  |  |  |  |  |  |  |  |  | E1 |
| Less than higher completed | 97 | 45.1 | ( 37.9, 53.4) |  | 45.4 | ( 38.6, 52.4) |  | 45.1 | ( 37.9, 53.4) |  |
| Higher complete | 80 | 32.5 | ( 25.6, 39.4) |  | 33.4 | ( 27.3, 39.4) |  | 32.4 | ( 25.6, 39.4) |  |
| Above higher | 49 | 22.4 | ( 16, 28.3) |  | 21.2 | ( 15.9, 26.5) |  | 22.3 | ( 16, 28.3) |  |
| No response | 1 | 0.0 | (0,0) |  | 0.0 | (0,0) |  | 0.2 | | |  |
| Main source of income/ employment |  |  |  |  |  |  |  |  |  | E1 |
| None | 111 | 52.1 | ( 43.5, 60.5) |  | 50.6 | ( 42.8, 58.5) |  | 52.0 | ( 43.4, 60.4) |  |
| Household/domestic | 0 | 0.0 | ( 0, 0) |  | 0.0 | ( 0, 0) |  | 0.0 | ( 0, 0) |  |
| Professional/businessman | 6 | 2.1 | ( 0.4, 4.3) |  | 2.1 | ( 0.4, 4.3) |  | 2.3 | ( 0.4, 4.3) |  |
| Employee | 16 | 5.4 | ( 2.6, 8.3) |  | 5.4 | ( 2.8, 8.2) |  | 5.7 | ( 2.6, 8.4) |  |
| Mechanic/factory worker/labourer | 4 | 1.5 | ( 0.2, 3.3) |  | 1.8 | ( 0.4, 3.9) |  | 1.7 | ( 0.2, 3.3) |  |
| Hairdresser/salon worker/shop/sales/service | 23 | 12.6 | ( 7.6, 17.4) |  | 13.0 | ( 1.4, 17.8) |  | 11.7 | ( 7.6, 17.5) |  |
| Taxi/bus/truck driver | 6 | 2.5 | ( 0.7, 4.8) |  | 2.9 | ( 0.9, 11.2) |  | 2.4 | ( 0.7, 4.8) |  |
| Watchman/security guard | 9 | 4.9 | ( 1.6, 9.4) |  | 5.7 | ( 1.8, 10.1) |  | 4.6 | ( 1.6, 9.4) |  |
| Hawker/street vendor/casual labourer | 47 | 17.9 | ( 13.2, 24) |  | 17.7 | ( 7.2, 22.4) |  | 18.3 | ( 13.2, 24) |  |
| Other | 5 | 1.0 | ( 0.1, 2.4) |  | 0.9 | ( 0.2, 16.2) |  | 1.3 | ( 0.1, 2.4) |  |
| HIV infection |  |  |  |  |  |  |  |  |  | E1 |
| Yes | 12 | 3.1 | ( 0.7, 6.9) |  | 3.7 | ( 0.8, 7.9) |  | 4.3 | ( 0.7, 7.2) |  |
| No | 215 | 96.9 | ( 93.1, 99.3) |  | 96.3 | ( 92.1, 99.2) |  | 95.7 | ( 92.8, 99.3) |  |
| Missing data | 0 | 0.0 | (0,0) |  | 0.0 | (0,0) |  | 0.0 | (0,0) |  |
| Hepatitis B infection |  |  |  |  |  |  |  |  |  | E1 |
| Yes | 7 | 2.9 | ( 0.9, 6.1) |  | 2.7 | ( 0.8, 4.7) |  | 3.0 | ( 0.9, 6.1) |  |
| No | 217 | 95.8 | ( 92.1, 98.2) |  | 96.4 | ( 94.2, 98.3) |  | 95.7 | ( 92.2, 98.2) |  |
| Missing data** | 3 | 1.2 | ( 0, 3.3) |  | 0.9 | ( 0, 2.2) |  | 1.3 | ( 0, 3.3) |  |
| Hepatitis C infection |  |  |  |  |  |  |  |  |  | E1 |
| Yes | 19 | 7.3 | ( 2.3, 13.7) |  | 8.4 | ( 2.9, 15.6) |  | 8.1 | ( 2.4, 13.9) |  |
| No | 205 | 91.4 | ( 84.8, 96.4) |  | 90.6 | ( 83.6, 96.3) |  | 90.6 | ( 84.6, 96.4) |  |
| Missing data** | 3 | 1.3 | ( 0, 3.4) |  | 0.9 | ( 0, 2.2) |  | 1.3 | ( 0, 3.4) |  |
| HIV/Hepatitis B co-infection |  |  |  |  |  |  |  |  |  | E1 |
| Yes | 0 | 0.0 | (0,0) |  | 0.0 | (0,0) |  | 0.0 | (0,0) |  |
| No | 224 | 98.7 | ( 96.5, 100) |  | 99.1 | ( 97.8, 100) |  | 98.7 | ( 96.6, 99.9) |  |
| Missing data** | 3 | 1.3 | ( 0, 3.5) |  | 0.9 | ( 0, 2.2) |  | 1.3 | ( 0.1, 3.4) |  |
| HIV/Hepatitis C co-infection |  |  |  |  |  |  |  |  |  | E2 |
| Yes | 10 | 2.1 | ( 0, 5.5) |  | 2.4 | ( 0, 6) |  | 3.7 | ( 0, 5.6) |  |
| No | 214 | 96.6 | ( 92.7, 99.1) |  | 96.7 | ( 0, 99) |  | 95.0 | ( 92.6, 99) |  |
| Missing data| | 3 | 1.3 | ( 0, 3.4) |  | 1.0 | ( 0, 98.8) |  | 1.3 | ( 0, 3.4) |  |

* Sample size n out of total of N=227 (seeds not included)

† Population estimates computed using RDSII/DA estimator method^1^ - same results as in manuscript table 1

‡ Population estimates computed using RDSI/DS estimator method^2^

^Population estimates computed using RDSII estimator method^1^

^|^ Insufficient data to compute reliable 95% CI (Confidence Interval)

**Data missing due to laboratory error

E1=Equilibrium reached at 1% level, E2=Equilibrium reached at 2% level

Table S2 – Socio-demographic characteristics and prevalence of HIV and other infections among FSW in Tripoli, Libya, 2010

| Sociodemographic indicators | n* | RDSII/DA estimate† | |  | RDS I/DS estimate‡ | |  | RDS II estimate^ | |  |
| --- | --- | --- | --- | --- | --- | --- | --- | --- | --- | --- |
|  |  | % | 95% CI |  | % | 95% CI |  | % | 95% CI |  |
| Age |  |  |  |  |  |  |  |  |  |  |
| 15-19 | 2 | 0.0 | | |  | 0.0 | | |  | 1.7 | | |  |
| 20-29 | 35 | 73.4 | | |  | 75.1 | ( 59.6, 86.4) |  | 68.8 | | |  |
| 30-39 | 19 | 17.7 | | |  | 15.3 | ( 8.1, 24.8) |  | 17.4 | | |  |
| 40-49 | 10 | 8.9 | | |  | 9.6 | ( 2.9, 19.4) |  | 9.5 | | |  |
| ≥ 50 | 3 | 0.0 | | |  | 0.0 | | |  | 2.7 | | |  |
| Civil status |  |  |  |  |  |  |  |  |  | E1 |
| Married, living with spouse | 1 | 2.9 | ( 0, 7) |  | 4.8 | ( 0, 13.7) |  | 2.2 | ( 0, 7) |  |
| Married, living with other sexual partner | 0 | 0.0 | ( 0, 0) |  | 0.0 | ( 0, 0) |  | 0.0 | ( 0, 0) |  |
| Married, not living with spouse/other sexual  partner | 4 | 4.6 | ( 0.9, 10.3) |  | 5.8 | ( 0, 10.5) |  | 4.1 | ( 0.9, 10.2) |  |
| Not married, living with sexual partner | 17 | 31.5 | ( 17.4, 44.2) |  | 33.2 | ( 2.7, 42.7) |  | 32.3 | ( 17.3, 44) |  |
| Not married, not living with sexual partner | 47 | 61.1 | ( 47.8, 76.7) |  | 56.2 | ( 25.9, 65.9) |  | 61.3 | ( 47.9, 76.6) |  |
| No response | 0 | 0.0 | ( 0, 0) |  | 0.0 | ( 0, 0) |  | 0.0 | ( 0, 0) |  |
| Country of origin |  |  |  |  |  |  |  |  |  | E1 |
| Libya | 8 | 12.4 | ( 0, 29.4) |  | 14.9 | ( 0, 35.4) |  | 12.4 | ( 0.8, 30.3) |  |
| Abroad | 61 | 87.6 | ( 70.5, 100) |  | 85.1 | ( 52.3, 98.1) |  | 87.6 | ( 69.7, 99.2) |  |
| Time period lived in Libya |  |  |  |  |  |  |  |  |  |  |
| <10 years | 48 | 82.7 | ( 62.6, 95.8) |  | 83.7 | ( 64.3, 95.8) |  | 80.1 | ( 62.5, 95.5) |  |
| 10-20 years | 11 | 4.6 | ( 0.7, 10.9) |  | 4.3 | ( 0.9, 9.6) |  | 6.7 | ( 0.8, 11) |  |
| >20 years | 10 | 12.7 | ( 1, 30.8) |  | 12.0 | ( 1.2, 29.8) |  | 13.3 | ( 1.3, 30.9) |  |
| Travelled for work for >1 month during past year | 8 | 20.5 | ( 4.1, 49.6) |  | 24.2 | ( 4.5, 49.9) |  | 9.7 | ( 4.3, 48.9) |  |
| Education level |  |  |  |  |  |  |  |  |  |  |
| Less than higher education completed | 49 | 81.3 | ( 69.5, 90.8) |  | 82.9 | ( 73.5, 90.4) |  | 79.0 | ( 69.8, 90.6) |  |
| Higher completed | 7 | 10.8 | ( 3.4, 21.3) |  | 10.7 | ( 3.8, 18.6) |  | 9.2 | ( 3.5, 21.2) |  |
| Above higher | 7 | 5.9 | ( 0.1, 13.2) |  | 3.5 | ( 0.7, 8.3) |  | 8.5 | ( 0.3, 13.3) |  |
| No response | 6 | 2.0 | ( 0, 6.5) |  | 2.9 | ( 0, 8.5) |  | 3.3 | ( 0, 6.7) |  |
| Main source of income other than sex work |  |  |  |  |  |  |  |  |  | E1 |
| None | 42 | 66.4 | ( 47.1, 82.8) |  | 61.9 | ( 44.6, 78.6) |  | 66.0 | ( 47, 82.6) |  |
| Household/domestic | 19 | 25.3 | ( 11.9, 44.1) |  | 24.4 | ( 12.1, 38.5) |  | 24.6 | ( 12, 44.3) |  |
| Professional/businessman | 1 | 0.0 | ( 0, 0) |  | 0.0 | ( 0, 0) |  | 1.0 | | |  |
| Employee | 0 | 0.0 | ( 0, 0) |  | 0.0 | ( 0, 0) |  | 0.0 | ( 0, 0) |  |
| Mechanic/factory worker/labourer | 0 | 0.0 | ( 0, 0) |  | 0.0 | ( 0, 0) |  | 0.0 | ( 0, 0) |  |
| Hairdresser/salon worker/shop/sales/service | 7 | 8.3 | ( 2.3, 13.6) |  | 13.6 | ( 4.1, 23.9) |  | 8.5 | ( 2.3, 13.7) |  |
| Taxi/bus/truck driver | 0 | 0.0 | ( 0, 0) |  | 0.0 | ( 0, 0) |  | 0.0 | ( 0, 0) |  |
| Watchman/security guard | 0 | 0.0 | ( 0, 0) |  | 0.0 | ( 0, 0) |  | 0.0 | ( 0, 0) |  |
| Hawker/street vendor/casual labourer | 0 | 0.0 | ( 0, 0) |  | 0.0 | ( 0, 0) |  | 0.0 | ( 0, 0) |  |
| Other | 0 | 0.0 | ( 0, 0) |  | 0.0 | ( 0, 0) |  | 0.0 | ( 0, 0) |  |
| HIV infection |  |  |  |  |  |  |  |  |  |  |
| Yes | 7 | 15.7 | ( 3.2, 32.6) |  | 20.6 | ( 4.7, 39.5) |  | 11.7 | ( 3.2, 32.9) |  |
| No | 61 | 83.7 | ( 66.7, 95.9) |  | 78.3 | ( 59.6, 94) |  | 87.6 | ( 66.6, 95.7) |  |
| Missing data** | 1 | 0.6 | ( 0, 2.6) |  | 1.1 | ( 0, 3.7) |  | 0.7 | ( 0, 2.6) |  |
| Hepatitis B infection |  |  |  |  |  |  |  |  |  |  |
| Yes | 2 | 0.0 | | |  | 0.0 | | |  | 4.5 | | |  |
| No | 67 | 100.0 | ( 100, 100) |  | 100.0 | ( 100, 100) |  | 95.5 | ( 100, 100) |  |
| Missing data | 0 | 0.0 | ( 0, 0) |  | 0.0 | ( 0, 0) |  | 0.0 | ( 0, 0) |  |
| Hepatitis C infection |  |  |  |  |  |  |  |  |  | E1 |
| Yes | 5 | 5.2 | ( 1.2, 12.2) |  | 8.5 | ( 1.8, 17.2) |  | 5.4 | ( 1.2, 12.3) |  |
| No | 64 | 94.8 | ( 87.8, 98.8) |  | 91.5 | ( 82.8, 98.2) |  | 94.6 | ( 87.7, 98.8) |  |
| Missing data | 0 | 0.0 | ( 0, 0) |  | 0.0 | ( 0, 0) |  | 0.0 | ( 0, 0) |  |
| HIV/Hepatitis B co-infection |  |  |  |  |  |  |  |  |  | E1 |
| Yes | 0 | 0.0 | ( 0, 0) |  | 0.0 | ( 0, 0) |  | 0.0 | ( 0, 0) |  |
| No | 68 | 99.3 | ( 97, 100) |  | 98.8 | ( 96.3, 100) |  | 99.3 | ( 97.1, 100) |  |
| Missing data** | 1 | 0.7 | ( 0, 3) |  | 1.2 | ( 0, 3.7) |  | 0.7 | ( 0, 2.9) |  |
| HIV/Hepatitis C co-infection |  |  |  |  |  |  |  |  |  | E1 |
| Yes | 3 | 3.7 | ( 0, 12.6) |  | 6.3 | ( 0, 18.7) |  | 2.8 | ( 0, 12.5) |  |
| No | 65 | 95.6 | ( 86.7, 100) |  | 92.5 | ( 0, 98.4) |  | 96.6 | ( 86.6, 99.3) |  |
| Missing data** | 1 | 0.7 | ( 0, 2.7) |  | 1.2 | ( 0, 98.8) |  | 0.7 | ( 0, 2.7) |  |

* Sample size n out of total of N=69 (seeds not included)

† Population estimates computed using RDSII/DA estimator method^1^ - same results as in manuscript table 1

‡ Population estimates computed using RDSI/DS estimator method^2^

^Population estimates computed using RDSII estimator method^1^

^|^ Insufficient data to compute reliable 95% CI (Confidence Interval)

** Data missing due to laboratory error

E1=Equilibrium reached at 1% level, E2=Equilibrium reached at 2% level

Table S3 – Sexual behaviour and risk factors for HIV infection among MSM in Tripoli, Libya, 2010

| Risk factors and other indicators | n* | RDSII/DA estimate† | |  | RDS I/DS estimate‡ | |  | RDS II estimate^ | |  |
| --- | --- | --- | --- | --- | --- | --- | --- | --- | --- | --- |
|  |  | % | 95% CI |  | % | 95% CI |  | % | 95% CI |  |
| Sexual behaviour with males |  |  |  |  |  |  |  |  |  |  |
| Age at first sexual intercourse |  |  |  |  |  |  |  |  |  | E1 |
| <18 years | 142 | 58.1 | ( 48.7, 67.2) |  | 56.7 | ( 48.2, 65.2) |  | 59.2 | ( 48.6, 67.1) |  |
| >= 18 years | 83 | 40.7 | ( 31.5, 50.1) |  | 42.2 | ( 33.7, 50.9) |  | 39.5 | ( 31.6, 50) |  |
| No response | 2 | 1.2 | ( 0, 3.8) |  | 1.0 | ( 0, 2.7) |  | 1.2 | ( 0, 3.8) |  |
| Forced sexual debut | 33 | 13.8 | ( 8, 19.1) |  | 12.1 | ( 8, 16.5) |  | 13.7 | ( 8, 19.1) | E1 |
| Was forced to sexual intercourse in past 12 months | 12 | 5.0 | ( 2.5, 8.8) |  | 5.5 | ( 2.8, 8.6) |  | 5.2 | ( 2.5, 8.8) | E1 |
| Anal sex with multiple partners (>1) in past 6 months | 207 | 87.5 | ( 81.1, 92.4) |  | 88.7 | ( 84.6, 93) |  | 87.3 | ( 81.1, 92.4) | E1 |
| Number of anal sex partners in past 6 months |  |  |  |  |  |  |  |  |  | E1 |
| ≤3 | 108 | 55.8 | ( 47.5, 63.9) |  | 56.7 | ( 49.2, 64) |  | 55.0 | ( 47.5, 63.9) |  |
| >3 | 119 | 44.2 | ( 36.1, 52.5) |  | 43.3 | ( 36, 50.8) |  | 45.0 | ( 36.1, 52.5) |  |
| Ever insertive anal sex | 223 | 98.0 | | |  | 97.2 | ( 92.2, 100) |  | 97.2 | | | E1 |
| Insertive anal sex during past 6 months | 220 | 97.6 | ( 94.7, 99) |  | 97.0 | ( 94.5, 98.9) |  | 97.6 | ( 94.7, 99) | E1 |
| Number of partners |  |  |  |  |  |  |  |  |  | E1 |
| 1 | 23/220 | 13.7 | (8.4,19.5) |  | 11.9 | (8,16) |  | 13.8 | (8.4,19.5) |  |
| 2 to 4 | 124/220 | 56.4 | (49.2,64.8) |  | 59.5 | (52.8,66.2) |  | 56.2 | (49.2,64.8) |  |
| >4 | 73/220 | 29.9 | (21.7,37.3) |  | 28.6 | (21.9,35.5) |  | 30 | (21.8,37.3) |  |
| Ever receptive anal sex | 22 | 6.3 | ( 2.1, 11.7) |  | 5.7 | ( 2, 10.6) |  | 8.2 | ( 2.2, 12) |  |
| Receptive anal sex during past 6 months | 17 | 3.8 | ( 1.1, 7.7) |  | 3.7 | ( 1.2, 7.4) |  | 5.1 | ( 1.1, 7.9) | E2 |
| Number of partners |  |  |  |  |  |  |  |  |  | E2 |
| 1 | 2/17 | 0.0 | | |  | 0.0 | | |  | 17.5 | | |  |
| 2 to 4 | 3/17 | 38.9 | ( 0, 91.1) |  | 41.2 | ( 0, 89.1) |  | 25.9 | ( 0, 87) |  |
| >4 | 11/17 | 56.6 | ( 4, 100) |  | 53.8 | ( 0, 81.1) |  | 51.8 | ( 11.5, 100) |  |
| don't know | 1/17 | 4.5 | ( 0, 17.8) |  | 4.9 | ( 0, 100) |  | 4.9 | ( 0, 20.5) |  |
| Ever had group sex | 42 | 16.9 | ( 10.9, 22.9) |  | 17.4 | ( 11.7, 23.5) |  | 17.7 | ( 10.9, 22.9) | E1 |
| Had group sex during past 6 months | 22 | 8.1 | ( 4.5, 12.4) |  | 8.5 | ( 5, 12.2) |  | 8.8 | ( 4.5, 12.4) | E2 |
| Number of partners |  |  |  |  |  |  |  |  |  | E1 |
| 2 to 5 | 19/22 | 87.2 | ( 66.2, 100) |  | 87.1 | ( 69.4, 100) |  | 86.6 | ( 66.3, 100) |  |
| 6 to 8 | 3/22 | 12.8 | ( 0, 33.8) |  | 12.9 | ( 0, 30.6) |  | 13.4 | ( 0, 33.7) |  |
| Condom use during last anal sex | 55/227 | 21.0 | ( 14.5, 27.7) |  | 20.2 | ( 14.5, 26.1) |  | 21.6 | ( 14.5, 27.7) | E1 |
| Condom break during anal sex in last month | 13/88 | 14.9 | ( 6.7, 22.6) |  | 14.9 | ( 8, 22.2) |  | 14.8 | ( 6.7, 22.6) | E1 |
| Ever used lubricants | 127 | 54.3 | ( 45.8, 61.8) |  | 52.0 | ( 44.8, 59.1) |  | 54.2 | ( 45.8, 61.8) | E1 |
| Type of lubricants commonly used |  |  |  |  |  |  |  |  |  | E1 |
| Water based | 1/127 | 0.6 | ( 0, 2.8) |  | 0.8 | ( 0, 2.4) |  | 0.5 | ( 0, 2.7) |  |
| Oil based | 46/127 | 39.3 | ( 30.3, 50.7) |  | 40.9 | ( 31.8, 49.7) |  | 39.1 | ( 30.3, 50.8) |  |
| No response | 80/127 | 60.1 | ( 48.5, 69.2) |  | 58.5 | ( 49.3, 67.3) |  | 60.3 | ( 48.5, 69.2) |  |
| Consistent lubricant use during past 6 month | 30/227 | 11.3 | ( 6.2, 16.5) |  | 11.7 | ( 7, 17.1) |  | 11.6 | ( 6.2, 16.5) | E1 |
| Had anal sexual intercourse with regular non-  commercial partner in past 6 months | 186 | 79.2 | ( 71.3, 85.7) |  | 80.0 | ( 73.8, 86) |  | 79.6 | ( 71.2, 85.7) | E1 |
| Used a condom at last sex | 48/186 | 21.8 | ( 14.4, 30) |  | 21.4 | ( 14.8, 28.9) |  | 22.9 | ( 14.4, 30) | E1 |
| Consistent condom use | 23/186 | 10.4 | ( 5.6, 15.8) |  | 10.3 | ( 6, 15.1) |  | 11.0 | ( 5.6, 15.8) | E1 |
| Had anal sexual intercourse with non-regular non-  commercial partner in past 6 months | 156 | 69.0 | ( 61.8, 76.7) |  | 68.8 | ( 62, 75.3) |  | 68.8 | ( 61.8, 76.6) | E1 |
| Used a condom at last sex | 38/156 | 20.5 | ( 13.4, 28.2) |  | 20.2 | ( 13.9, 26.9) |  | 20.5 | ( 13.4, 28.2) | E1 |
| Consistent condom use | 28/156 | 15.8 | ( 9.8, 23.6) |  | 15.4 | ( 9.9, 21.5) |  | 16.1 | ( 9.8, 23.6) | E1 |
| Had anal sexual intercourse with commercial partner  in past 6 months | 69 | 26.5 | ( 19.3, 33.9) |  | 25.5 | ( 19.5, 31.9) |  | 27.2 | ( 19.4, 33.9) | E1 |
| Used a condom at last sex | 20/69 | 19.4 | ( 10.1, 32.2) |  | 20.6 | ( 11, 32) |  | 21.3 | ( 10.1, 32.2) | E1 |
| Consistent condom use | 13/69 | 13.7 | ( 6, 25.6) |  | 14.4 | ( 6.2, 24.1) |  | 14.8 | ( 6, 25.8) | E1 |
| Had oral sex (with any partner) during past 6 months | 75 | 30.3 | ( 22.9, 37.4) |  | 28.3 | ( 22.2, 34.7) |  | 30.8 | ( 22.9, 37.4) | E1 |
| Used a condom at last sex | 12/75 | 11.9 | ( 5.2, 21.3) |  | 12.8 | ( 6, 21) |  | 12.2 | ( 5.2, 21.3) | E1 |
| Consistent condom use | 8/75 | 7.2 | ( 2.3, 13.5) |  | 6.8 | ( 2.7, 11.9) |  | 7.1 | ( 2.3, 13.4) | E1 |
| Ejaculated in partner's mouth or partner ejaculated  in his mouth | 33/75 | 45.2 | ( 32.1, 58.9) |  | 44.9 | ( 33.5, 56) |  | 46.2 | ( 32.1, 58.9) | E1 |
| Sexual behaviour with females |  |  |  |  |  |  |  |  |  |  |
| Ever had sex with female partner | 159 | 68.5 | ( 59.7, 77) |  | 66.7 | ( 58.3, 74.8) |  | 69.2 | ( 59.7, 77) | E1 |
| Age at first sexual intercourse |  |  |  |  |  |  |  |  |  |  |
| <18 | 76/159 | 44.3 | ( 34, 54.9) |  | 45.9 | ( 36.2, 55.2) |  | 45.6 | ( 34, 54.9) | E1 |
| >=18 | 77/159 | 51.8 | ( 41.4, 61.4) |  | 50.5 | ( 41.5, 59.6) |  | 50.9 | ( 41.4, 61.4) |  |
| no response | 6/159 | 4.0 | ( 1.2, 7.7) |  | 3.6 | ( 1.2, 6.4) |  | 3.5 | ( 1.2, 7.7) |  |
| Men who have risky sex with men and women** | 93 | 38.5 | ( 30.6, 46.5) |  | 38.2 | ( 31.2, 45.4) |  | 38.7 | ( 30.6, 46.5) | E1 |
| Consumed alcohol ≥ 4 times per week in past 6 months | 7 | 3.7 | ( 1.4, 6.8) |  | 4.4 | ( 1.9, 7.3) |  | 3.6 | ( 1.4, 6.7) | E1 |
| Non-injecting drug use in past 6 months | 77 | 29.5 | ( 21.1, 38.3) |  | 30.0 | ( 22.1, 38.7) |  | 30.1 | ( 21.1, 38.3) | E1 |
| Ever injected drugs | 11 | 4.0 | ( 1, 8.6) |  | 4.7 | ( 1.3, 9.3) |  | 4.0 | ( 1.1, 8.9) | E1 |
| Age of first injection |  |  |  |  |  |  |  |  |  | E1 |
| <21 | 1/11 | 0.0 | ( 0, 0) |  | 0.0 | ( 0, 0) |  | 0.0 | ( 0, 0) |  |
| >=21 | 10/11 | 100.0 | ( 100, 100) |  | 100.0 | ( 100, 100) |  | 92.8 | | |  |
| Frequency of injection drug use during past 6 months |  |  |  |  |  |  |  |  |  | E1 |
| ≤ once per week | 1/11 | 7.2 | ( 0, 43.1) |  | 8.5 | ( 0, 45.5) |  | 7.2 | ( 0, 43.1) |  |
| > once per week | 10/11 | 92.8 | ( 55.9, 100) |  | 91.5 | ( 0, 95.1) |  | 92.8 | ( 56.2, 100) |  |
| Shared needle/syringe at last injection | 2/11 | 0.0 | | |  | 0.0 | | |  | 0.0 | | | E1 |
| Perception of risk for HIV infection |  |  |  |  |  |  |  |  |  | E1 |
| High risk | 17 | 5.7 | ( 3.2, 9.7) |  | 6.8 | ( 3.8, 10.4) |  | 6.1 | ( 3.2, 9.7) |  |
| Medium risk | 42 | 19.5 | ( 13.9, 26.1) |  | 20.6 | ( 15.1, 26.5) |  | 19.3 | ( 13.9, 26.1) |  |
| Low risk | 33 | 15.4 | ( 10.2, 20.8) |  | 14.3 | ( 10.1, 18.7) |  | 15.0 | ( 10.2, 20.8) |  |
| No risk | 115 | 49.8 | ( 41.1, 57.1) |  | 47.7 | ( 40.8, 54.7) |  | 51.0 | ( 41.1, 57.1) |  |
| No response | 20 | 9.6 | ( 5.6, 14.4) |  | 10.7 | ( 6.4, 15.2) |  | 8.7 | ( 5.6, 14.4) |  |

* Sample size n out of total of N=227 where not indicated otherwise (seeds not included)

† Population estimates computed using RDSII/DA estimator method^1^ - same results as in manuscript table 2

‡ Population estimates computed using RDSI/DS estimator method^2^

^Population estimates computed using RDSII estimator method^1^

^|^ Insufficient data to compute reliable 95% CI (Confidence Interval)

**Respondents who had unprotected sex with a woman at least once in the last 6 months, and who have had unprotected anal sex with at least one other man in the last 6 months

Table S4 – Sexual behaviour and risk factors for HIV infection among FSW in Tripoli, Libya, 2010

| Risk factors and other indicators | n* | RDSII/DA estimate† | |  | RDS I/DS estimate‡ | |  | RDS II estimate^ | |  |
| --- | --- | --- | --- | --- | --- | --- | --- | --- | --- | --- |
|  |  | % | 95% CI |  | % | 95% CI |  | % | 95% CI |  |
| Age at first sexual intercourse |  |  |  |  |  |  |  |  |  | E1 |
| <18 | 20 | 22.7 | ( 10.1, 37) |  | 18.8 | ( 10.5, 28.9) |  | 22.5 | ( 10.2, 37.3) |  |
| ≥18 | 48 | 75.5 | ( 58.9, 89.1) |  | 78.2 | ( 64.3, 89) |  | 75.8 | ( 58.5, 88.7) |  |
| No response | 1 | 1.8 | ( 0, 7.3) |  | 3.0 | ( 0, 10.3) |  | 1.7 | ( 0, 7.4) |  |
| Age at first selling sex |  |  |  |  |  |  |  |  |  |  |
| <18 | 5 | 2.4 | ( 0, 7.9) |  | 2.6 | ( 0, 6.8) |  | 2.4 | ( 0.2, 8) |  |
| ≥18 | 63 | 96.2 | ( 89.7, 99.6) |  | 95.2 | ( 0, 99.5) |  | 96.2 | ( 89.6, 99.3) |  |
| no response | 1 | 1.3 | ( 0, 5.3) |  | 2.2 | ( 0, 10.7) |  | 1.3 | ( 0, 5.3) |  |
| Was forced to sexual intercourse in past 12 months | 13 | 18.2 | ( 8.4, 33.4) |  | 20.7 | ( 10.3, 32.3) |  | 17.1 | ( 8.5, 33.5) | E1 |
| Reasons for exchanging money or goods for sex |  |  |  |  |  |  |  |  |  | E1 |
| economic | 58 | 90.1 | ( 80.1, 96.4) |  | 86.9 | ( 76.7, 94.9) |  | 87.3 | ( 79.9, 96.3) |  |
| forced | 4 | 3.8 | ( 0.8, 7.8) |  | 6.4156 | ( 1.5, 12.3) |  | 3.5 | ( 0.8, 7.8) |  |
| abandoned by family/husband | 6 | 6.1 | ( 1.2, 14.4) |  | 6.7072 | ( 1.1, 13.5) |  | 5.9 | ( 1.3, 14.6) |  |
| other | 1 | 0.0 | (0,0) |  | 0 | (0,0) |  | 3.4 | | |  |
| Main place where respondent met clients during past month |  |  |  |  |  |  |  |  |  |  |
| Own home | 14 | 14.6 | ( 0.9, 32.3) |  | 9.8 | ( 1.2, 22) |  | 23.7 | ( 1.2, 32.1) |  |
| Friend's home | 9 | 10.8 | ( 4, 25) |  | 7.7 | ( 2.1, 14.2) |  | 9.0 | ( 4.1, 24.9) |  |
| Sex partner's home | 10 | 11.3 | ( 2.5, 22.4) |  | 12.5 | ( 3.1, 23) |  | 13.2 | ( 2.7, 22.4) |  |
| On the streets | 8 | 20.0 | ( 4, 36.8) |  | 23.5 | ( 5.2, 42.2) |  | 11.3 | ( 4.1, 35.9) |  |
| By telephone | 5 | 6.0 | ( 0.6, 12.1) |  | 6.6 | ( 1.3, 14.3) |  | 6.0 | ( 0.7, 12) |  |
| In Café | 8 | 15.6 | ( 2.9, 36.2) |  | 15.4 | ( 2.1, 31.3) |  | 8.4 | ( 3, 34.8) |  |
| Brothel/ Connection house | 6 | 8.6 | ( 0, 23.9) |  | 6.8 | ( 0, 18.5) |  | 11.9 | ( 0, 23.8) |  |
| Other | 6 | 10.2 | ( 1.7, 18) |  | 13.2 | ( 0, 23.4) |  | 11.4 | ( 1.8, 17.9) |  |
| No response | 2 | 2.1 | ( 0, 7.6) |  | 3.2 | ( 0, 13.2) |  | 3.4 | ( 0, 7.7) |  |
| Missing data | 1 | 0.8 | ( 0, 4.1) |  | 1.2 | ( 0, 5.5) |  | 1.7 | ( 0, 4) |  |
| Total number of sexual partners in last six months |  |  |  |  |  |  |  |  |  |  |
| <10 | 26 | 25.9 | ( 15, 40.6) |  | 33.6 | ( 20.8, 47.1) |  | 29.1 | ( 15.2, 40.6) |  |
| 10 to 50 | 23 | 42.8 | ( 24.8, 57.8) |  | 30.1 | ( 19, 41.7) |  | 43.5 | ( 24.9, 57.8) |  |
| ≥50 | 17 | 30.5 | ( 16.1, 47.6) |  | 35.0 | ( 19.7, 50.8) |  | 25.9 | ( 16.2, 47.5) |  |
| don't know | 3 | 0.9 | ( 0, 3.2) |  | 1.4 | ( 0, 4.6) |  | 1.5 | ( 0, 3.3) |  |
| Number of regular clients in last six months |  |  |  |  |  |  |  |  |  |  |
| <10 | 9 | 22.6 | ( 5.7, 36.7) |  | 23.3 | ( 9.2, 38.8) |  | 19.3 | ( 5.8, 36.7) |  |
| 10 to 50 | 12 | 10.8 | ( 1.8, 24.6) |  | 9.5 | ( 2.1, 20.5) |  | 15.6 | ( 1.8, 24.6) |  |
| ≥50 | 18 | 21.4 | ( 12.5, 34.8) |  | 25.0 | ( 14.7, 36.6) |  | 20.8 | ( 12.5, 34.8) |  |
| don't know | 30 | 45.2 | ( 30.6, 62.5) |  | 42.3 | ( 29.4, 55.7) |  | 44.2 | ( 30.5, 62.4) |  |
| Number of one-time clients in last six months |  |  |  |  |  |  |  |  |  | E1 |
| <10 | 56 | 85.2 | ( 73.2, 90.3) |  | 83.3 | ( 75.8, 90.4) |  | 86.0 | ( 73.3, 90.3) |  |
| 10 to 50 | 6 | 8.3 | ( 3.2, 17.7) |  | 11.2 | ( 3.6, 18.6) |  | 7.6 | ( 3.2, 17.5) |  |
| ≥50 | 3 | 3.6 | ( 0, 10.9) |  | 1.9 | ( 0, 4.2) |  | 3.3 | ( 0.1, 10.8) |  |
| don't know | 4 | 2.9 | ( 0, 7.6) |  | 3.7 | ( 0, 8.2) |  | 3.0 | ( 0.3, 7.6) |  |
| Had sex with one-time client | 36 | 51.8 | ( 37.9, 69.3) |  | 51.6 | ( 38.7, 64.5) |  | 51.6 | ( 37.9, 69.3) | E1 |
| Used condom consistently | 24/36 | 63.4 | ( 41.4, 81.7) |  | 59.1 | ( 42.4, 78) |  | 64.0 | ( 41.3, 81.6) | E1 |
| Used a condom at last sex | 29/36 | 83.1 | ( 66.5, 95.4) |  | 84.4 | ( 72.1, 94.8) |  | 84.2 | ( 66.5, 95.4) | E1 |
| Reasons for not using a condom at last sex** |  |  |  |  |  |  |  |  |  | E1 |
| Not available | 1/36 | 1.1 | ( 0, 4.8) |  | 2.0 | ( 0, 6) |  | 1.1 | ( 0, 4.7) |  |
| Not pleasurable for client | 1/36 | 0.0 | ( 0, 0) |  | 0.0 | ( 0, 0) |  | 0.2 | | |  |
| Didn't think of it | 1/36 | 0.0 | ( 0, 0) |  | 0.0 | ( 0, 0) |  | 2.2 | | |  |
| Condom negotiation at last intercourse with one-time  client |  |  |  |  |  | ( 0, 0) |  |  | ( 0, 0) | E1 |
| Respondent suggested condom use | 26/36 | 54.2 | ( 31.3, 81.8) |  | 48.8 | ( 27.5, 88.1) |  | 58.8 | ( 31.2, 80.7) |  |
| Client suggested condom use | 3/36 | 17.3 | ( 4.3, 31.4) |  | 22.0 | ( 6.5, 44.1) |  | 13.0 | ( 4.6, 31.1) |  |
| Joint decision | 1/36 | 11.6 | ( 0, 33.3) |  | 16.3 | ( 0, 40.3) |  | 13.0 | ( 0, 32.7) |  |
| Not applicable/no response | 6/36 | 16.9 | ( 3.7, 32.3) |  | 12.9 | ( 0, 22.2) |  | 15.2 | ( 3.9, 32.3) |  |
| Had sex with regular client | 38 | 54.3 | ( 37.5, 70.3) |  | 55.0 | ( 41.3, 68.6) |  | 55.1 | ( 37.5, 70.3) | E1 |
| Used condom consistently | 22/38 | 56.8 | ( 39.2, 74.3) |  | 53.1 | ( 39.1, 66.9) |  | 55.7 | ( 39.3, 74.3) | E1 |
| Used a condom at last sex | 27/38 | 76.7 | ( 61.1, 88.7) |  | 73.2 | ( 60.5, 85.1) |  | 76.4 | ( 61.2, 88.7) | E1 |
| Reasons for not using a condom with last regular client** |  |  |  |  |  |  |  |  |  | E1 |
| Not available | 2/38 | 3.3 | ( 0, 10.2) |  | 5.2 | ( 0, 12.7) |  | 3.4 | ( 0, 10.1) |  |
| Not pleasurable for respondent | 2/38 | 2.7 | ( 0, 8.2) |  | 4.4 | ( 0, 10.8) |  | 2.6 | ( 0, 8.1) |  |
| Not pleasurable for client | 8/38 | 5.1 | ( 2, 11.6) |  | 5.9 | ( 2.2, 10.1) |  | 5.1 | ( 2, 11.6) |  |
| Didn't think of it | 1/38 | 0.0 | ( 0, 0) |  | 0.0 | ( 0, 0) |  | 2.2 | | |  |
| Trust partner | 4/38 | 3.0 | ( 0.6, 9.4) |  | 4.4 | ( 1, 8.8) |  | 2.9 | ( 0.7, 9.3) |  |
| No response | 1/38 | 0.0 | ( 0, 0) |  | 0.0 | ( 0, 0) |  | 1.1 | | |  |
| Condom negotiation at last intercourse with regular client |  |  |  |  |  | ( 0, 0) |  |  | ( 0, 0) | E1 |
| Respondent suggested condom use | 24/38 | 71.8 | ( 46.7, 78.8) |  | 65.1 | ( 49.6, 100) |  | 68.3 | ( 46.7, 78.4) |  |
| Client suggested condom use | 2/38 | 0.0 | | |  | 0.0 | | |  | 5.1 | | |  |
| Joint decision | 1/38 | 3.9 | ( 0, 11.9) |  | 6.9 | ( 0, 21.6) |  | 3.0 | ( 0, 11.9) |  |
| Not applicable/no response | 11/38 | 24.3 | ( 18.1, 48.7) |  | 28.0 | ( 0, 38.4) |  | 23.6 | ( 18.1, 48.5) |  |
| Condom use during anal and dry sex |  |  |  |  |  | ( 0, 0) |  |  | ( 0, 0) |  |
| Ever had anal sex | 2 | 1.5 | ( 0, 4.4) |  | 2.3 | ( 0, 5.6) |  | 1.4 | ( 0, 4.3) | E1 |
| Had anal sex in last 30 days and used condom at last anal  sex | 0/1 | 0 | ( 0, 0) |  | 0 |  |  | 0 | ( 0, 0) | E1 |
| Ever had dry sex | 19 | 33.9 | ( 17.3, 48.8) |  | 23.2 | ( 14.3, 33.8) |  | 33.9 | ( 17.2, 48.8) | E1 |
| Had dry sex in last 30 days and used condom at last dry sex | 8/12 | 67.4 | ( 29.1, 94.6) |  | 39.0 | ( 18.9, 75.7) |  | 66.8 | ( 29.3, 93.7) | E1 |
| Reported STI symptom (unusual genital discharge, ulcer or sore) during last year | 21 | 27.8 | ( 15, 42.2) |  | 38.0 | ( 23.7, 51.6) |  | 28.8 | ( 15, 42.3) | E2 |
| Correct action taken for STI †† | 7/21 | 24.3 | ( 8.8, 51.2) |  | 23.4 | ( 8, 43.1) |  | 25.3 | ( 8.8, 51.4) | E2 |
| Consumed alcohol ≥ 4 times per week in past 6 months | 3 | 0.0 | | |  | 0.0 | | |  | 6.7 | | | E1 |
| Non-injecting drug use in past 6 months | 1 | 1.2 | ( 0, 4.3) |  | 2.1 | ( 0, 6.1) |  | 1.1 | ( 0, 4.3) | E1 |
| Ever injected drugs | 2 | 0.0 | | |  | 0.0 | | |  | 1.6 | | |  |

* Sample size n out of total of N=69 where not indicated otherwise (seeds not included)

† Population estimates computed using RDSII/DA estimator method^1^ - same results as in manuscript table 3

‡ Population estimates computed using RDSI/DS estimator method^2^

^Population estimates computed using RDSII estimator method^1^

^|^ Insufficient data to compute reliable 95% CI (Confidence Interval)

**Multiple answers possible

†† Took at least two of the following actions: sought care at public health facility, sough care at private health facility, told sex partner about symptoms, stopped having sex, used condom during sex

E1=Equilibrium reached at 1% level, E2=Equilibrium reached at 2% level

Insufficient data to compute CI

Table S5 – Access to services, knowledge and attitudes related to HIV among MSM in Tripoli, Libya, 2010

| Indicators | n* | RDSII/DA estimate† | |  | RDS I/DS estimate‡ | |  | RDS II estimate^ | |  |
| --- | --- | --- | --- | --- | --- | --- | --- | --- | --- | --- |
|  |  | % | 95% CI |  | % | 95% CI |  | % | 95% CI |  |
| Exposure to HIV prevention programmes ** | 3 | 0.9 | ( 0, 2.3) |  | 1.2 | ( 0, 2.8) |  | 0.9 | ( 0, 2.3) | E1 |
| Condom use |  |  |  |  |  |  |  |  |  |  |
| Respondents with condoms to hand †† | 1 | 0.0 | ( 0, 0) |  | 0.0 | ( 0, 0) |  | 0.4 | ( 0, 0.5) | E1 |
| Knowledge of correct condom use | 31 | 12.1 | ( 7.4, 16.5) |  | 11.5 | ( 7.5, 15.6) |  | 12.3 | ( 7.4, 16.5) | E1 |
| Knowledge of how to obtain condoms | 214 | 94.6 | ( 91.2, 97.2) |  | 93.6 | ( 90.2, 96.9) |  | 94.4 | ( 91.2, 97.2) | E1 |
| Knowledge of where to get lubricants | 177 | 75.7 | ( 69.7, 81.9) |  | 76.0 | ( 70.5, 81.2) |  | 75.7 | ( 69.7, 81.9) | E1 |
| HIV testing |  |  |  |  |  |  |  |  |  |  |
| Ever voluntarily received HIV test and knows results ‡‡ | 104 | 44.8 | ( 38.3, 52.5) |  | 45.7 | ( 39.7, 51.7) |  | 45.0 | ( 38.3, 52.5) | E1 |
| Underwent an HIV test in past 12 months, and knows  results | 106 | 45.6 | ( 38.7, 53.5) |  | 46.7 | ( 40.4, 52.9) |  | 45.7 | ( 38.7, 53.5) | E1 |
| HIV-related knowledge |  |  |  |  |  |  |  |  |  |  |
| Correctly identified ways to prevent sexual transmission of  HIV and who reject major misconceptions about HIV  Transmission ^^ | 42 | 16.8 | ( 11.6, 22.7) |  | 18.5 | ( 13.3, 24.1) |  | 17.1 | ( 11.6, 22.6) | E1 |
| STI-related knowledge: Correctly identified at least two common signs/symptoms of STIs in both men and women | 3 | 1.3 | ( 0, 3.5) |  | 1.6 | ( 0, 3.9) |  | 1.3 | ( 0.3, 3.5) | E1 |
| Has been arrested during past 12 months | 23 | 10.9 | ( 6.4, 16.9) |  | 9.0 | ( 5.8, 12.7) |  | 10.9 | ( 6.4, 16.9) | E1 |
| Main reason for arrest |  |  |  |  |  |  |  |  |  | E1 |
| Being on/ possession of drugs | 2/23 | 4.6 | ( 0, 15.5) |  | 4.8 | ( 0, 14) |  | 4.6 | ( 0, 15.5) |  |
| Being drunk | 4/23 | 13.4 | ( 0, 32.1) |  | 17.4 | ( 0, 34.6) |  | 15.5 | ( 1.4, 32.2) |  |
| Fight | 10/23 | 46.2 | ( 21.1, 73.9) |  | 48.0 | ( 0, 68.9) |  | 42.8 | ( 21.1, 73.8) |  |
| Exchanging sex for money | 0/23 | 0.0 | ( 0, 0) |  | 0.0 | ( 0, 0) |  | 0.0 | ( 0, 0) |  |
| Other | 6/23 | 35.8 | ( 9, 59.8) |  | 29.7 | ( 10.3, 89.1) |  | 35.8 | ( 9, 59.5) |  |
| No response | 1/23 | 0.0 | ( 0, 0) |  | 0.0 | ( 0, 0) |  | 1.2 | | |  |
| Stigma & discrimination |  |  |  |  |  |  |  |  |  |  |
| Absence of stigma towards people living with HIV || | 31 | 13.1 | ( 8.8, 19.5) |  | 14.4 | ( 9.6, 20) |  | 12.9 | ( 8.8, 19.5) | E1 |
| Has been refused different services in the last 12 months  because he is believed to have sex with men *** | 12 | 5.2 | ( 1.9, 10.3) |  | 4.1 | ( 1.8, 6.7) |  | 5.4 | ( 1.9, 10.3) | E1 |
| At least one verbal insult experienced by respondent in the  last 12 months because he is believed to have sex with men | 24 | 9.1 | ( 5.6, 13.9) |  | 9.6 | ( 6, 13.7) |  | 9.1 | ( 5.6, 14) | E1 |
| Has been hit, kicked, or beaten in the last 12 months  because he is believed to have sex with men | 2 | 0.8 | ( 0, 2.2) |  | 0.6 | ( 0, 1.4) |  | 0.8 | ( 0, 2.2) | E1 |

* Sample size n out of total of N=227 where not indicated otherwise (seeds not included)

† Population estimates computed using RDSII/DA estimator method^1^ - same results as in manuscript table 4

‡ Population estimates computed using RDSI/DS estimator method^2^

^Population estimates computed using RDSII estimator method^1^

^|^ Insufficient data to compute CI

** Respondents who know where they can get tested for HIV and who have been given condoms through outreach service, drop-in centre or health facility in past 12 months

†† Respondents who could show at least one condom to interviewer

‡‡ "tested voluntarily" means that respondents were not directly be forced to be tested (but potentially indirectly if health certificate is required)

^^ Respondents who know that a healthy-looking person can transmit HIV, that the transmission risk can be reduced by having sex with only one faithful, uninfected partner, and by using condoms, and who reject the misconceptions that HIV can be transmitted by sharing a meal with someone infected and through mosquito bites.

^||^ Respondents who would be willing to share a meal with a person who has HIV or AIDS, would be willing to care at their house for a male or female relative who is ill with HIV, who would buy food from a shopkeeper or food seller who has HIV, who thinks a student infected with HIV, but is not sick with AIDS should be allowed to attend school, and a teacher who is infected with HIV, but is not sick should be allowed to continue to teach.

*** Respondents who have been refused health care, employment, education, restaurant service or police assistance

E1=Equilibrium reached at 1% level, E2=Equilibrium reached at 2% level

Table S6 – Access to services, knowledge and attitudes related to HIV among FSW in Tripoli, Libya, 2010

| Indicators | n* | RDSII/DA estimate† | |  | RDS I/DS estimate‡ | |  | RDS II estimate^ | |  |
| --- | --- | --- | --- | --- | --- | --- | --- | --- | --- | --- |
|  |  | % | 95% CI |  | % | 95% CI |  | % | 95% CI |  |
| Exposure to HIV prevention programmes | | 0 | 0.0 | ( 0, 0) |  | 0.0 | ( 0, 0) |  | 0.0 | ( 0, 0) | E1 |
| Condom access and use |  |  |  |  |  |  |  |  |  |  |
| Respondents with condoms to hand** | 2 | 1.1 | ( 0, 3.5) |  | 1.9 | ( 0, 4.9) |  | 1.1 | ( 0, 3.5) | E1 |
| Knowledge of correct condom use | 34 | 49.2 | ( 34.3, 64.6) |  | 43.6 | ( 31.7, 55.7) |  | 49.6 | ( 34.3, 64.6) | E1 |
| Knowledge of how to obtain condoms | 57 | 81.7 | ( 69, 90.2) |  | 74.0 | ( 62.5, 86.1) |  | 81.3 | ( 69.1, 90.2) | E1 |
| Knowledge of place to get female condoms | 9 | 8.2 | ( 1.2, 17.9) |  | 11.4 | ( 2.1, 22.7) |  | 12.3 | ( 1.4, 18.3) |  |
| (Knowledge of where to get lubricants) |  |  |  |  |  |  |  |  |  |  |
| HIV testing |  |  |  |  |  |  |  |  |  |  |
| Ever received HIV test | 52 | 65.1 | ( 48.8, 83.1) |  | 61.3 | ( 47, 78.4) |  | 63.2 | ( 48.7, 83) | E2 |
| Underwent an HIV test in past 12 months and knows results | 32 | 38.6 | ( 25.1, 55.3) |  | 40.4 | ( 28.3, 53.6) |  | 38.8 | ( 25.1, 55.4) | E1 |
| Underwent an HIV test in past 12 months, received pre-test  counselling and knows results | 7 | 9.0 | ( 2.8, 17.2) |  | 11.4 | ( 5.1, 18.5) |  | 9.2 | ( 2.8, 17.2) | E1 |
| HIV-related knowledge |  |  |  |  |  |  |  |  |  |  |
| Correctly identified ways to prevent sexual transmission of  HIV and who reject major misconceptions about HIV  transmission†† | 16 | 18.6 | ( 10.1, 34.9) |  | 16.3 | ( 8.7, 25.6) |  | 18.4 | ( 10.2, 35) | E1 |
| Knowledge of mother to child transmission | 4 | 3.3 | ( 0.6, 8.1) |  | 5.0 | ( 1.3, 10.5) |  | 3.4 | ( 0.7, 8.1) | E1 |
| STI-related knowledge: Correctly identified at least two common signs/symptoms of STIs in both men and women | 7 | 5.4 | ( 1.5, 14.6) |  | 8.3 | ( 2.3, 16.6) |  | 5.4 | ( 1.5, 14.8) | E1 |
| Has been arrested during past 12 months | 3 | 5.9 | ( 0, 12.1) |  | 8.1 | ( 0, 17) |  | 5.6 | ( 0.4, 12) | E1 |
| Main reason for arrest |  |  |  |  |  |  |  |  |  | E1 |
| Being on/ in possession of drugs | 0 | 0.0 | ( 0, 0) |  | 0.0 | ( 0, 0) |  | 0.0 | ( 0, 0) |  |
| Being drunk | 0 | 0.0 | ( 0, 0) |  | 0.0 | ( 0, 0) |  | 0.0 | ( 0, 0) |  |
| Fight | 0 | 0.0 | ( 0, 0) |  | 0.0 | ( 0, 0) |  | 0.0 | ( 0, 0) |  |
| Exchanging sex for money | 1 | 0.0 | ( 0, 0) |  | 0.0 | ( 0, 0) |  | 0.0 | ( 0, 0) |  |
| Other | 1 | 0.0 | ( 0, 0) |  | 0.0 | ( 0, 0) |  | 0.0 | ( 0, 0) |  |
| No response | 0 | 0.0 | ( 0, 0) |  | 0.0 | ( 0, 0) |  | 0.0 | ( 0, 0) |  |
| Stigma & discrimination |  |  |  |  |  |  |  |  |  |  |
| Absence of stigma towards people living with HIV ‡‡ | 16 | 26.2 | ( 11.8, 41.9) |  | 23.9 | ( 12, 38.2) |  | 23.6 | ( 11.9, 42.1) |  |
| Has been refused different services in the last 12 months  because someone believed she is FSW|| | 9 | 9.8 | ( 2.9, 18.6) |  | 10.7 | ( 4.6, 18.4) |  | 10.0 | ( 2.9, 18.7) | E1 |
| At least one verbal insult experienced by respondent in the  last 12 months because someone believed she is a FSW | 22 | 28.3 | ( 15.8, 40.5) |  | 29.6 | ( 18.9, 41) |  | 28.4 | ( 15.8, 40.5) | E1 |
| Hit, kicked or beaten in last 12 months because someone  believed she is a FSW | 6 | 11.0 | ( 3.4, 20.6) |  | 12.9 | ( 4.6, 21.7) |  | 11.3 | ( 3.4, 20.5) | E1 |

* Sample size n out of total of N=227 where not indicated otherwise (seeds not included

† Population estimates computed using RDSII/DA estimator method^1^ - same results as in manuscript table 4

‡ Population estimates computed using RDSI/DS estimator method^2^

^Population estimates computed using RDSII estimator method^1^

^|^ Respondents who know where they can get tested for HIV and who have been given condoms through outreach service, drop-in centre or health facility in past 12 months

** Respondents who could show at least one condom to interviewer

†† Respondents who know that a healthy-looking person can transmit HIV, that the transmission risk can be reduced by having sex with only one faithful, uninfected partner, and by using condoms, and who reject the misconceptions that HIV can be transmitted by sharing a meal with someone infected and through mosquito bites.

‡‡ Respondents who would be willing to share a meal with a person who has HIV or AIDS, would be willing to care at their house for a male or female relative who is ill with HIV, who would buy food from a shopkeeper or food seller who has HIV, who thinks a student infected with HIV, but is not sick with AIDS should be allowed to attend school, and a teacher who is infected with HIV, but is not sick should be allowed to continue to teach.

^||^ Respondents who have been refused health care, employment, education, restaurant service or police assistance

E1=Equilibrium reached at 1% level, E2=Equilibrium reached at 2% level

**References:**

1. Volz E, Heckathorn DD. Probability based estimation theory for respondent driven sampling. *Journal of Official Statistics.* 2008;24(1):79.

2. Heckathorn DD. Respondent-driven sampling II: deriving valid population estimates from chain-referral samples of hidden populations. *Soc Probl.* 2002;49:11-34.
